# Deep learning forecasting of large induced earthquakes via precursory signals

**DOI:** 10.1038/s41598-024-52935-2

**Published:** 2024-02-05

**Authors:** Vincenzo Convertito, Fabio Giampaolo, Ortensia Amoroso, Francesco Piccialli

**Affiliations:** 1 Istituto Nazionale di Geofisica e Vulcanologia, Osservatorio Vesuviano, Naples, Italy; 2 Department of Mathematics and Applications “R. Caccioppoli”, Univeristy of Naples Federico II, Naples, Italy; 3 Department of Physics “E.R. Caianiello”, University of Salerno, Fisciano, SA Italy

**Keywords:** Geophysics, Seismology

## Abstract

Precursory phenomena to earthquakes have always attracted researchers’ attention. Among the most investigated precursors, foreshocks play a key role. However, their prompt identification with respect to background seismicity still remains an issue. The task is worsened when dealing with low-magnitude earthquakes. Despite that, seismology and, in particular real-time seismology, can nowadays benefit from the use of Artificial Intelligence (AI) to face the challenge of effective precursory signals discrimination. Here, we propose a deep learning method named PreD-Net (precursor detection network) to address precursory signal identification of induced earthquakes. PreD-Net has been trained on data related to three different induced seismicity areas, namely The Geysers, located in California, USA, Cooper Basin, Australia, Hengill in Iceland. The network shows a suitable model generalization, providing considerable results on samples that were not used during the network training phase of all the sites. Tests on related samples of induced large events, with the addition of data collected from the Basel catalogue, Switzerland, assess the possibility of building a real-time warning strategy to be used to avoid adverse consequences during field operations.

## Introduction

Deterministic earthquake prediction is still far from being possible due to the complexity and the limited knowledge of the system geoscientists have to deal with. However, considerable steps have been made toward the identification of reliable precursory phenomena that can allow us to understand if the system is evolving toward a critical/unstable state. Although their proper identification is still debated, foreshocks have been referred to as the most obvious premonitory phenomenon preceding earthquakes^1^ thus representing the most promising candidate^2,3^. Foreshocks have been interpreted as the failing of populations of small patches of fault as they reach a critical stress that eventually but not necessarily become large earthquakes^4^ or as a part of the nucleation process which ultimately leads to the mainshock^5,6^.

Distinguishing precursors, such as foreshocks from ordinary seismic activities, as for example earthquake swarms or switching, is not trivial and may hamper their usefulness in reliable earthquake prediction^3,7,8^. In practice, identification of the precursory phase of large earthquakes is mainly based on the analysis of earthquake catalogues and more recently from the analysis of geodetic data, and from waveform similarity analysis^9^. As an example, timely and accurate earthquake location can help to envisage earthquakes space migration. Besides, seismic catalogues of tectonic earthquakes allow investigating statistical features that characterise foreshocks with respect to mainshocks and aftershocks. For instance, changes in the slope of the Gutenberg-Richter relation^10^, namely the b-value, or power-law time-to-failure fitting have been analyzed as discrimination tools^5,11^. Whatever the adopted tool, all the implemented approaches require empirical criteria for selecting time and space windows and seismicity occurrence models, such as time-dependent Poisson^12^ or ETAS model^13,14^, to identify groups of earthquakes as candidates for being classified as foreshocks. Nevertheless, the significant amount of data collected in the last years and the advances in computer hardware have given Artificial Intelligence (AI) a great popularity in almost all scientific areas, including seismology.

AI techniques can learn significant patterns from the data to generate models to support and sustain human expertise. In particular, different approaches from Machine Learning (ML) and Deep Learning (DL) are successfully applied in the study of earthquakes^15–18^ and their detection^19–21^, with also some application in the field of induced seismicity^22^, and particular aimed at trying to anticipate the location of the areas where earthquakes are expected to occur^23^. According to^24^ earthquake catalogues collected by using AI have reached an unprecedented quality and detail that can help seismologists formulate and test new hypotheses about precursors of large earthquakes.

In this study, we propose a strategy for the identification of precursors of the large induced earthquake in a sequence based on a supervised DL approach that can be implemented in real-time applications. It should be noted that the use of induced seismicity^25^ brings with it the intrinsic difficulty that earthquakes may not occur as foreshock/mainshock/aftershock sequences but rather may occur as sequences of earthquakes with magnitudes close to each other. However, the choice to analyse induced seismicity was guided by the fact that seismic catalogues of induced earthquakes in the magnitude range of interest for the present study are characterised by a larger number of events and a lower minimum magnitude of completeness compared to tectonic seismicity. This is a key point since, at least for tectonic earthquakes, seismic catalogue incompleteness can produce artefacts in observed rates of both foreshocks and aftershocks^14^ making the statistical approach ineffective. In addition, the time and space evolution of the seismicity can be correlated to known field operations.

We investigated a set of specific features, which are generally considered prognostic of the earthquake preparatory phase (i.e. the minimum magnitude of completeness Mc, the b-value, moment magnitude ($M_W$), the moment rate, duration of events’ group, the coefficient of variation CoV , the Fractal Dimension, the Nearest-Neighbour distance, the Shannon’s Information Entropy and associated uncertainties). We analysed data collected in three regions of induced earthquakes, namely The Geysers (TG) geothermal field^26,27^, Cooper Basin (CB) geothermal reservoir^28,29^, and the Hengill geothermal field (HG)^30^. Moreover, to further assess the generalization capabilities of the approach, it has been tested on a stand-alone series, that is, not used for training and validation, extracted from the Basel (BS) catalogue, related to injection operations in 2006. The proposed DL approach relies on a three-step system: data labelling; a Neural Network (NN), namely the *PreD-Net*, for classification; and finally, a warning strategy.

In the data labelling step, a label is associated with each observation (i.e. each earthquake), either 0 or 1. Label 1 identifies a potential precursor, while label 0 is for background seismicity or events following the largest earthquake in the sequence (see "Methods"). So, the precursor recognition problem is recast to a binary classification problem. The proposed *PreD-Net* is a Neural Network made up of three types of layers: convolutional, recurrent and dense. There are other approaches in seismology literature exploiting similar architectures. For example, Convolutional Neural Networks^31,32^ are widely used in seismology, as for the Recurrent^33,34^ and Dense^35,36^ Networks. Furthermore, it is also possible to find networks made up of different layers, such as Convolutional and Dense^37,38^ or Convolutional and Recurrent^21,39^. Finally, architectures made up of both Convolutional, Dense networks and Recurrent networks have been discussed in several practical applications^40^.

In order to explore the feasibility of a real-time application of the proposed methodology, we implemented a probabilistic warning system, resembling the traffic light system^11,41,42^, that operates using the *PreD-Net* predictions. At each of the three colours of the traffic light, green, orange and red, a different level of warning, respectively no alarm, a soft alarm and a strong alarm is associated.

Here we show that AI can effectively discriminate precursors of the largest event in a sequence with respect to background seismicity once properly trained. Furthermore, the proposed warning strategy can provide valuable alerts up to hours before the occurrence of a potentially damaging earthquake, allowing us to make prompt decisions about field operations.

## Results

### Study areas and data preparation

**Figure 1:**
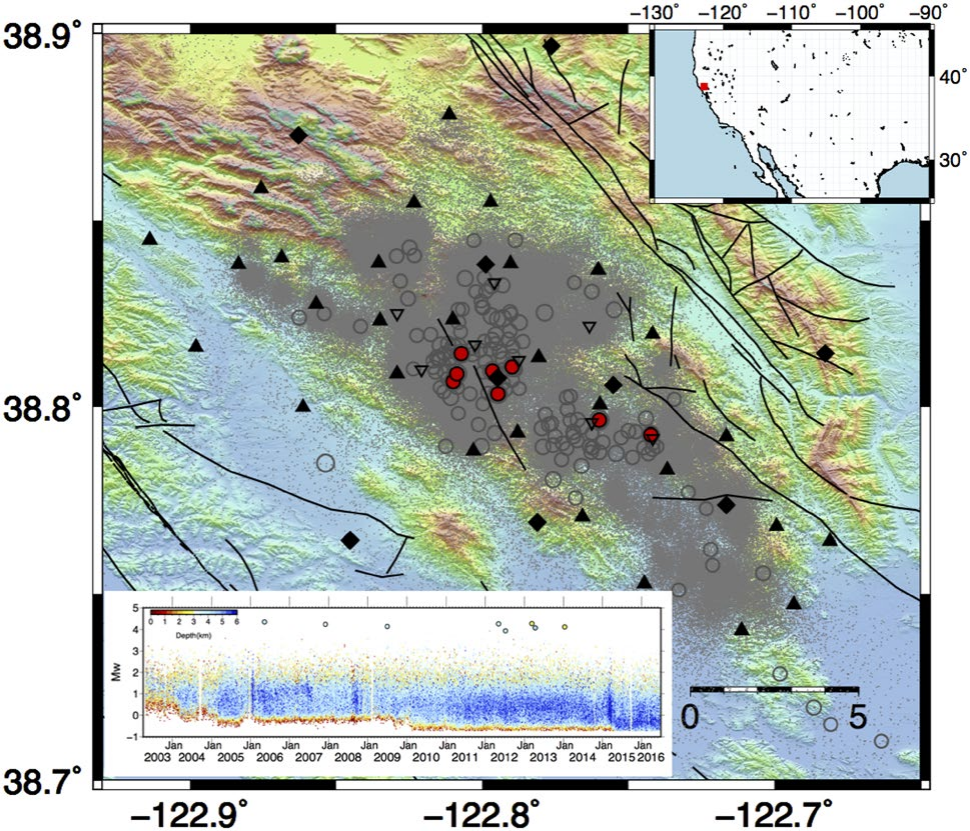
Characteristics of seismicity at The Geysers geothermal area. The epicentral distribution of the earthquakes is represented with dots and circles: light grey dots indicate earthquakes with $M_W$ less than 3, and grey circle earthquakes with $M_W$ larger or equal to 3. The larger events with $M_W$ greater than 3.9 are marked with red dots. The upper inset shows the location map of the Geyser geothermal area. The lower inset shows the magnitude temporal distribution, the symbols are coloured according to the depth. Black diamonds and triangles represent the location of seismic stations. Reverse empty triangles represent the wells’ position. The figures are generated by using the version 5 of Generic Mapping Tools (https://www.generic-mapping-tools.org/).

The Geysers (TG) is a vapour-dominated geothermal field located 120 km north of San Francisco (Fig. 1) and is the most productive geothermal area in the world^43^. Commercial exploitation, which began in 1960, has resulted in increased seismicity — due to fluids injection and extraction — concentrated in the first 6 km near the production and injection wells. We analysed data collected by a seismic network maintained by the Lawrence Berkeley National Laboratory Calpine (BG) and by the Northern California Seismic Network (NCSN). The analysis is focused on seismicity recorded from 2003 to 2016, which includes about 450000 events with magnitude $M_W$ ranging between $-0.7$ and 4.3.

**Figure 2:**
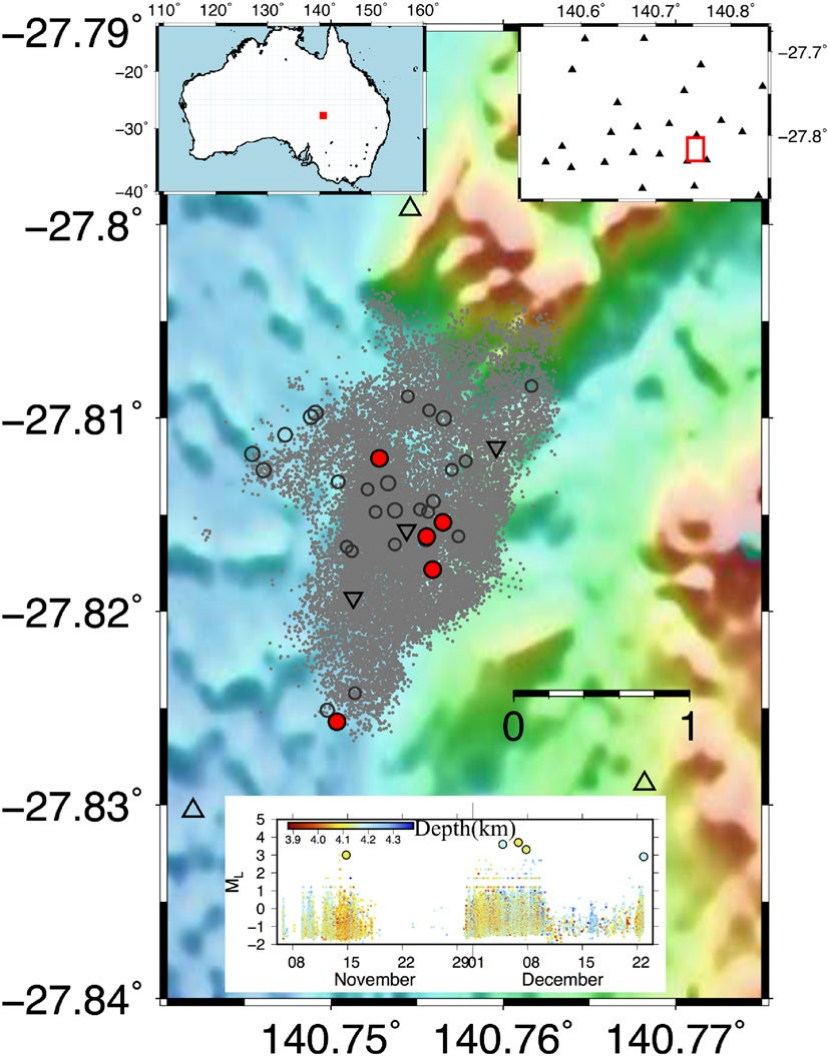
Characteristics of seismicity at Cooper Basin geothermal area. The epicentral distribution of the earthquakes is represented with a circle: grey dots represent earthquakes with $M_L$ less than 2, grey circles $M_L$ greater or equal to 2, and finally the main events with $M_L$ greater than 2.9 are marked with red dots. The two upper insets show the location map of the Cooper Basin geothermal area and the seismic monitoring network, respectively. The lower inset shows the magnitude temporal distribution, the symbols are coloured according to the depth. The reverse triangle represents the well’s position. The figures are generated by using the version 5 of Generic Mapping Tools (https://www.generic-mapping-tools.org/).

The Cooper Basin (CB) geothermal field is situated in the northeast of South Australia (Fig. 2). Field operations aimed at exploiting geothermal resources started in 2002. Since then, a total of six deep wells have been drilled in three distinct fields: the Habanero, the Jolokia and the Savina. In the present study, we referred to the Habanero field where stimulation activities finalised to enhance the hydraulic conductivity in the subsurface were conducted. These operations were accompanied by conspicuous seismic activity^44,45^. The seismic catalogue used in this study consists of 23285 seismic events recorded from November 2003 to December 2003 with magnitude $M_L$ ranging between − 2.0 and 3.7.

**Figure 3:**
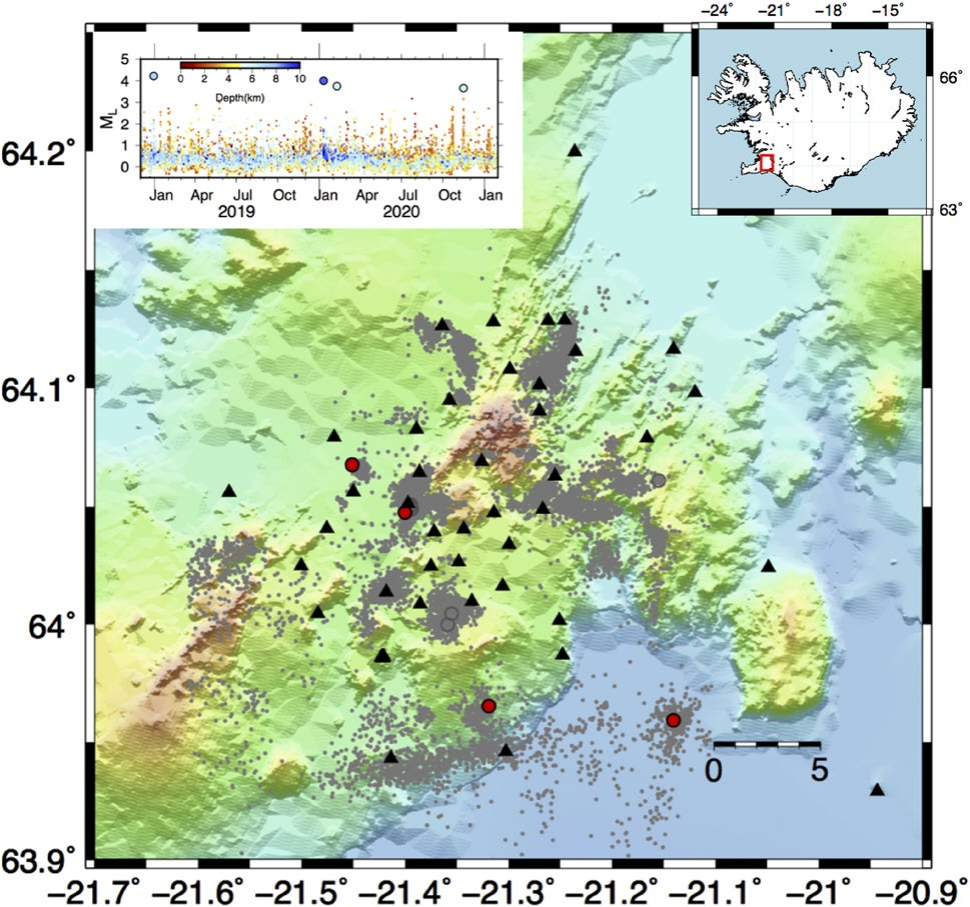
Characteristics of seismicity at Hengill geothermal area. The epicentral distribution of the earthquakes is represented with a circle: grey dots represent earthquakes with $M_L$ less than 3, grey circle $M_L$ greater or equal to 3, and finally the main events with $M_L$ greater than 3.5 are marked with red dots. The two upper insets show the location map of the Hengill geothermal area and the magnitude temporal distribution, the symbols are coloured according to the depth. The figures are generated by using the version 5 of Generic Mapping Tools (https://www.generic-mapping-tools.org/).

The Hengill (HG) geothermal field is located in southwest Iceland (Fig. 3) on the boundary between the North American and Eurasian plates. Geothermal energy exploitation started in the late 1960s for electrical power and heat production. The two biggest geothermal power plants in Iceland, Nesjavellir and Hellisheidi, are located in the Hengill region. Overall, the number of wells in Nesjavellir and Hellisheidi is 76 with a maximum depth of 2 km. Seismic events were already observed during the first drilling and testing phases of the boreholes^46^ and increased with the increasing number of the wells and the injection operations^30^. In the present study, we analysed earthquakes recorded from 2018/12/01 to 2021/01/31 collected during the COntrol SEISmicity and Manage Induced earthQuakes (COSEISMIQ) project, thanks to which the available number of stations has increased to 44^30^. The total number of earthquakes is 15318 with magnitude $M_L$ ranging between $-0.6$ and 4.2. The application to the Hengill geothermal field is noteworthy since the corresponding dataset contains both induced and natural seismicity^30^.

**Figure 4:**
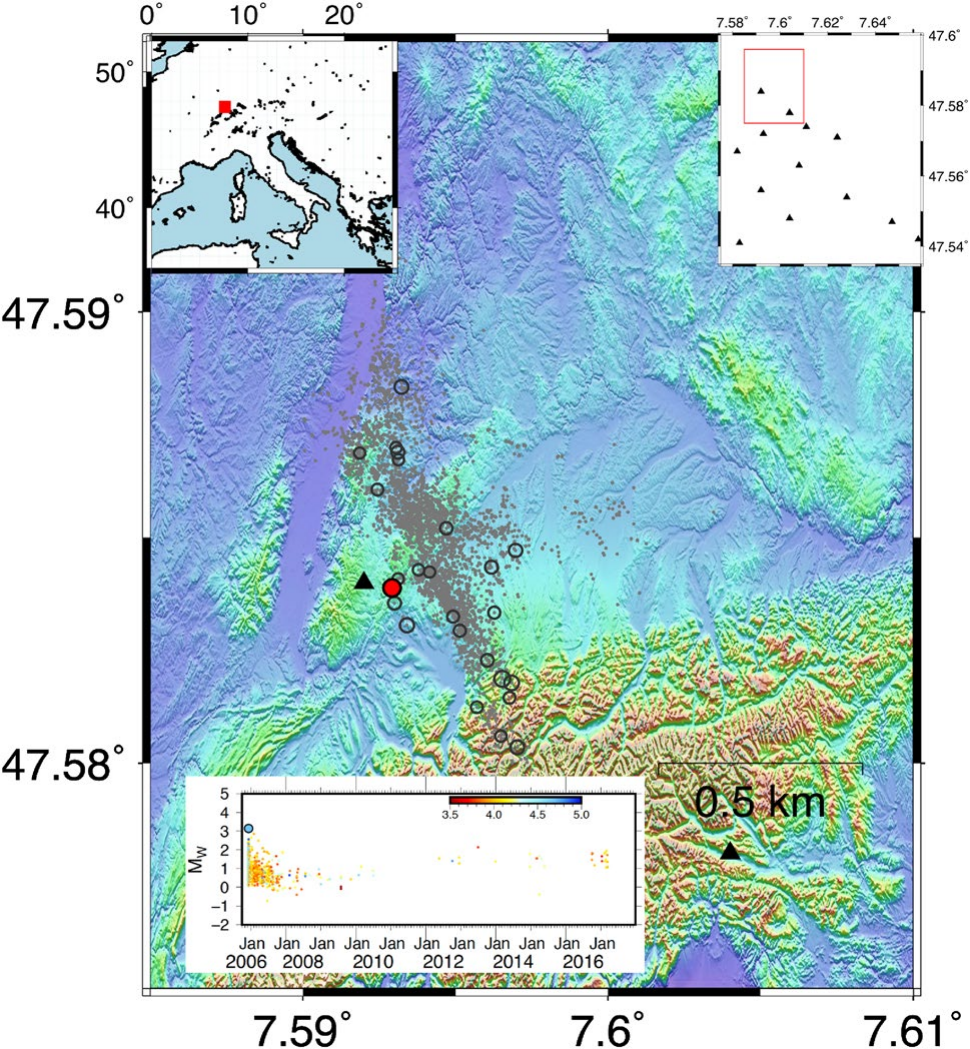
Characteristics of seismicity at Basel geothermal area. The epicentral distribution of the earthquakes is represented with a circle: grey dots represent earthquakes with $M_W$ less than 2, grey circles $M_W$ greater or equal to 2, and finally, the event with $M_W$ 3.1 is with red dots. The two upper insets show the location map of the Basel geothermal area and the seismic monitoring network. The lower inset shows the magnitude temporal distribution, the symbols are coloured according to the depth. The figures are generated by using the version 5 of Generic Mapping Tools (https://www.generic-mapping-tools.org/).

The Basel (BS) geothermal field is located in Switzerland and it was designed to provide thermal and electrical energy to Basel city (Fig. 4). BS represents an example of an Enhanced Geothermal System (EGS) in which high-pressure fluids have been injected into sub-soil to increase the permeability of the medium and facilitate fluid circulation. The project started with a stimulation phase on 2 December 2006. The injection was stopped on 8 December 2006 when a $M_L$ 3.7 ($M_W$ 3.1) occurred and the project was definitively suspended in April 2011^47^. In the present study, we analyzed 3684 earthquakes with magnitude $M_W$ ranging between $-{\textbf {2.4}}$ and 3.1.

With the aim of designing a precursor detection strategy, 16 statistical variables (i.e. the minimum magnitude of completeness Mc, the b-value, moment magnitude ($M_W$), the moment rate, events’ group duration, the coefficient of variation CoV , the Fractal Dimension, the Nearest-Neighbour distance, the Shannon’s Information Entropy, and associated uncertainties) have been calculated for the earthquakes of the first three seismic catalogues; then, for each catalogue, the larger magnitude events have been identified and related time series have been extracted. It is worth underlining that the term “time series” refers to a predetermined number of sequential events — 2000 for TG and CB geothermal areas or 600 for HG — taken in the neighbourhood of the earthquake of interest. This selection is coherent with that used to forecast labquakes (earthquakes generated during fracture experiments) by using Deep Learning^48^. As for the fourth catalogue, the same features have been extracted, and a series of 2000 elements-akin to what was done for TG and CB-has been designated as an additional test to assess the model’s generalization capabilities. Importantly, since no samples from BS were utilized during the training phase, our objective is to determine whether the model can discern common patterns characteristic of the preparatory phase leading up to the large earthquake in the induced seismic sequence, even if involving characteristics of the geothermal region that have not been encountered during training.

**Figure 5:**
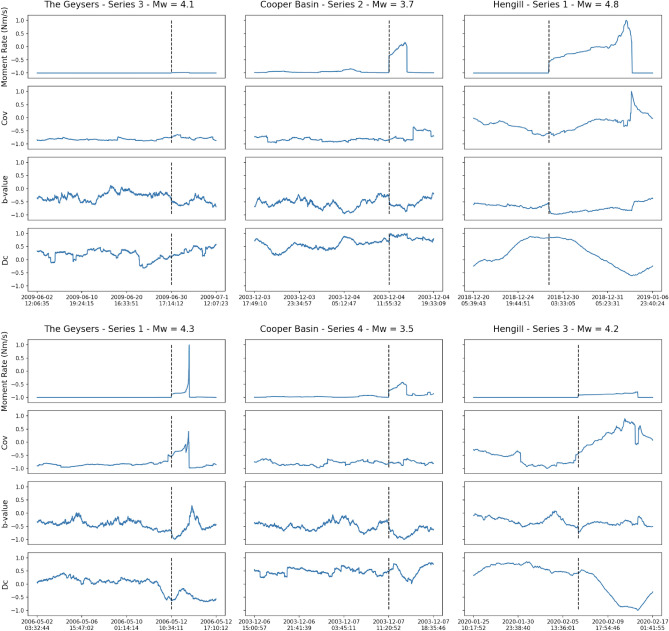
Time series of selected statistical variables. Trend of four statistical variables analysed for the training data (in the upper panels) and for the testing ones (lower panels). All the quantities have been normalised. The vertical black dashed lines mark the time of the largest event in the sequence whose magnitude is shown in the upper part of each panel. From top to bottom: moment rate, coefficient of variation (CoV), b-value, and fractal dimension (Dc).

Overall, the final dataset consists of the collection of seismic events — also referred to as samples — related to the 16 larger earthquakes: 8 for TG, 5 for CB, and 3 for HG, with the addition of a series from BS used solely for testing purposes.

Each sample is a vector of 16 statistical variables considered as features for the precursors’ identification process. A label, set to 0 for background seismicity and 1 for potential precursors, has been assigned to each sample according to the space-time location of the corresponding earthquake with respect to the largest earthquake (see "Methods" section for details). Considering the sequence of events (the time series), candidates for a precursory phase of the largest earthquake are identified by evaluating their coherence with a given space-time region determined by exploiting a circular source rupture model and the $\beta$-statistic, as described in the Sect. "Methods". Figure 5 shows the behaviour of four features among the computed ones for six time series belonging to the study areas. An important characteristic that emerges from the figure and from the additional examples reported in the Supplementary Materials, is that under no circumstances all the features, at the same time, follow a trend that can clearly identify the occurrence of precursors. For example, as for the $b$-value, which is one of the most investigated features in these types of studies, we see that it does not always tend to decrease sharply before the main event. On the other hand, to support the decision of the network, there should be another feature among those analysed by the model that presents a specific trend as observed prior to large earthquakes. This is the case, for example, of the fractal dimension $D_c$, which is proportional to $b$-value^49^ or the moment rate that is expected to accelerate before a large earthquake^50^. We believe that the fact of considering and being able to analyse several distinct statistical variables, feasible perhaps only with the aid of AI, is a relevant point of this study.

### PreD-net training

The neural network for precursors’ identification has been designed according to the choice of framing the problem as a binary classification task. In particular, the aim is to provide a prediction of being an element of the background seismicity rather than a precursor for the generic sample. In this perspective, from the dataset used for training (TG, CB, and HG), the events related to the three largest earthquakes, i.e. three time series—one for each geothermal area—have been kept aside to form a *Test set*, on which the performances of the neural architecture will be assessed.

The samples of the remaining time series have been then split into a *Training set*, used for the learning phase of the model, and a *Validation set*, used to optimise the hyperparameters of the network. Specifically, 50%, randomly chosen, have been picked for the *Validation set* and 50% for the *Training set*. From this splitting strategy a consideration arises: during the learning procedure, due to the way the *Training set* is built, the neural network is trained on data representing both the classes (0 for the background seismicity, 1 for precursors) for the samples belonging to the 13 series of the *Training/Validation sets*. In this perspective, predictions on samples of the *Validation set*, which are in any case unseen by the model during the training phase, are made on contexts (small groups of samples coherent with respect to the label in the features’ space; see *Discussion* and “Data exploration and discussion about accuracy results” in Supplementary Materials for details) the network is somehow aware of. On the other hand, the classification of samples belonging to the *Test set* mimics a real-scenario application case, in which predicted labels are provided on completely unseen, in the learning stage, data.

It is worth anticipating that *PreD-Net*, for the aim of the present study, operates the classification in an element-wise manner: to each sample, which as mentioned above is a 16-dimensional vector, the network assigns a probability of belonging to the background class, which includes also precursors or subsequent events to the largest earthquake in the sequence. To avoid possible data leakage due to the sequentiality of the events, information about positioning in the temporal direction of the samples has been removed and a random shuffling has been applied before the *Train/Validation* split procedure (see “Data exploration and discussion about accuracy results” in Supplementary Materials). Removing the temporal order information and perturbing the sequencing of the samples prevent the classifier from just identifying the largest event position and consequently assigning the label precursor to all the earlier samples in a certain time window. Anyway, the causality of the earthquakes is preserved thanks to the fact that features are computed on backward temporal windows (see "Methods").

The *Validation set* is also useful to set the thresholds used in the warning strategy. This is based on a traffic light system constructed starting from the probabilities predicted by the *PreD-Net*. Then, the Cumulative Sum (CDF) of the probabilities is computed, and the CDF Differentiated (CDFD) is used as a signal. Each time there is a cross between the CDFD and two fixed thresholds, an alert is issued. The value of these thresholds is empirically set according to the results observed in the *Validation set*.

The training process of the *PreD-Net*, whose structure is discussed in section “Further details of PreD-Net architecture and training procedure” of the supplementary materials, is carried out on a Nvidia RTX 3090 in less than 20 minutes, while the entire testing of the warning procedure requires approximately 250ms to elaborate the test series.

### PreD-Net classification results

Table 1 and Fig. 11 show the results of the classification obtained by the *PreD-Net* on the *Validation set*. As can be observed, the model exhibits a notable accuracy in discriminating the precursors. It is worth underlining that, as pointed out before, the model has been trained on samples taken from both the precursors and background regions/events of each of the series, obviously except the ones constituting the *Test set*. In this sense, the model had the opportunity to understand patterns among the features that distinguish the two different classes for each seismic sequence of interest. This leads to an accuracy of about 0.98 in recognizing both classes of earthquakes, as shown in Table 1.

**Table 1:** Metric results about PreD-Net.

	Accuracy	Precision	Recall	F1-score	AUC
Validation - TG	0.985	0.984	0.983	0.984	0.972
Validation - CB	0.981	0.985	0.979	0.982	0.985
Validation - HG	0.980	0.987	0.980	0.983	0.969
Validation - total	0.987	0.984	0.985	0.985	0.977
Test - TG	0.921	0.926	0.924	0.923	0.817
Test - CB	0.832	0.852	0.819	0.831	0.773
Test - HG	0.843	0.829	0.803	0.817	0.762
Test - BL	0.783	0.782	0.783	0.765	0.684
Test - total	0.845	0.851	0.838	0.839	0.758

Generalisation capabilities of the *PreD-Net* are shown in Fig. 12, where the classification of background seismicity and precursors occurs on samples of the *Test set*, i.e. events belonging to the three time series kept aside before the *Train/Validation* splitting of the dataset: precursors are adequately predicted by the proposed model, with respect to the original labelling. In particular, a very precise prediction can be observed for the first series coming from the TG dataset (left panel of Fig. 12).

In the case of the series extracted from the CB dataset, a large uncertainty can be observed in the precursors predictions, in particular in certain temporal intervals after the start of the precursory phase. This is probably connected to the fact that fewer training points are present for this second geothermal region. In fact, when the samples belong to series that are completely unknown, i.e. no generic knowledge about the context the samples are drawn from is present, more difficulties arise in distinguishing precursors samples in the sequence. However, as can be observed in the right panel of Fig. 12, predicted precursors preserve the coherence with the ground truth, allowing to identify precursory events of the largest earthquake.

As for the test series extracted from the Hengill geothermal field catalogue (upper left panel in Fig. 12), a high coherence between the prediction and the temporal zone in which the events following the largest earthquake occur can be observed: the principal incongruencies concerns the classification of background samples as precursors, especially after the beginning of such preliminary phase. It is worth underlining that related to this geothermal area there are not only fewer series, but also fewer samples per series with respect to the other areas, which translates into a relatively small amount of training samples: however, also in this case the overall coherence between actual labels and predicted ones turns out to be high, confirming the generalisation abilities of the proposed neural model.

When it comes to the test series extracted from the BS catalogue, it’s crucial to highlight that the training set contains no samples from this geothermal field. Despite this, the model’s performance in predicting precursors was notably commendable. In this context, while the model preemptively identified precursors, there were some misclassifications for the events following the largest event. However, the confidence and accuracy levels of these predictions remain high, corroborating the efficacy of our approach.

In all the test cases, as well as for the validation samples, the consistency of the prediction with real labels of the data allows to develop a warning strategy, as described in Section Methodology, which in principle can also exploit real-time predictions (as demonstrated in the cases of test series) to provide alerts whenever precursors are identified. Moreover, as can be observed, the warning strategy accomplishes its task: it is able to provide prompt alarms by timely understanding that an earthquake is about to occur. The advance of the system to the earthquake of interest is about 24 hours for the TG test, 12 hours for the CB, and a few days for HG.

## Discussion

The identification of the precursors is among the most debated topics in seismology and the task is much more arduous for small-to-moderate magnitude earthquakes^2,3^. Induced seismicity, generally characterized by seismic catalogues with a lower minimum magnitude of completeness compared to tectonic earthquakes, may represent a natural laboratory to test procedures aimed at identifying the preparatory phase of a large earthquake. Our results raise the possibility of effectively identifying precursors of induced earthquakes in a sequence by analysing several statistical features, which are considered prognostic, with the aid of artificial intelligence. We found that the implemented NN, named *PreD-Net*, is able to identify patterns in the event-related features that would have been unlikely to be identified by a human operator. In fact, the NN can analyse all the features at the same time and assign to each one, and to each partial combination of them, a degree of relevance based on what it has learned during the training phase. On the other hand, a human operator may have a problem deciding which features are more prognostic and also how many of the features should exceed a given threshold before identifying the occurrence of a large earthquake. From Fig. 5, it is possible to understand the capability of the *PreD-Net* in finding complex links among the features. In fact, while some patterns are clearly recognizable, other ones, which represent more complex nonlinear relationships, are difficult to identify. Instead, the *PreD-Net* is able to handle the nonlinearity and to extract valuable information also in the most challenging situations.

In Fig. 11 we can observe the behaviour of the network in the classification of the samples belonging to the Validation set. The remarkable accuracy can be attributed to the Train/Validation split operated on the dataset. This conclusion is supported by further analyses conducted on the data distribution (see “Data exploration and discussion about accuracy results” in Supplementary Materials): for each time series related to an event of interest, projected samples are distributed in small groups, which are generally coherent with respect to the labelling.

**Figure 6:**
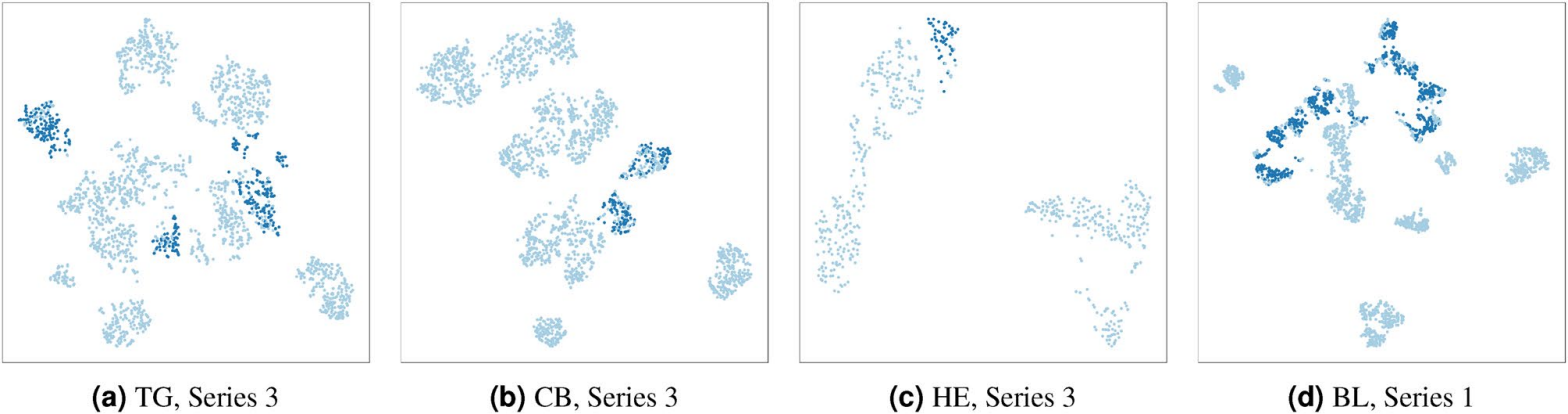
t-SNE analysis: Background samples represented by light blue dots and foreshock samples represented by dark blue dots, as displayed in the t-SNE representation of a sample series for each of the geothermal areas considered in this study.

These small clusters of events, as shown in Fig. 6, can be referred to as contexts, as regards the *Training set*, from which training samples are taken. Even if each validation sample is itself unseen, the context from which it is drawn can be inferred from the *Training set*. On the other hand, test samples are completely unknown from both the point of view of the samples and the contexts. The classification of these events must rely on generalisation capabilities of the model only, and this also motivates the different degree of confidence of the predictions, as discussed in the following. From Fig. 12 and Table 1 we can observe that the accuracy of the predictions on the *Test set* depends on the considered geothermal area. In particular, the classification metrics, based on Precision and Recall, on the TG events are considerably better than those on the CB and HG ones, and the warning strategy is more reliable too. This is probably due to the strong difference between the number of TG and CB/HG samples considered in the training step: as remarked before, from the training data the network learns different contexts, i.e. regions of the features’ space in which background events and precursors are located; of course, as the number of these contexts grows in the training phase, the neural model acquires better generalisation abilities. However, since intrinsic differences in the physics characteristics of the considered geothermal regions can exist, patterns learned for a geothermal area are not necessarily fully consistent for all the geothermal areas, and this implies that the classifier has more difficulties in predicting CB/HG unseen samples than TG ones. In conclusion, it is essential to highlight the dataset’s imbalance, which is clearly manifested in the superior performance metrics achieved by The Geysers in our results. Nevertheless, the notable performance observed with other test data implies that the model is capable of discerning underlying patterns within the dynamics under investigation. This lends support to the hypothesis that certain behaviors within sequences of induced seismicity are intrinsic, irrespective of their origins. In the context of this discussion, the results demonstrated by the model on the series from BS provide a significant point for consideration. Despite the absence of training samples from this geothermal region, meaning that the model has no prior context related to the Basel dataset, the discrimination between precursors and background seismicity still exhibits a commendable degree of reliability. This might be attributed to potential similarities - both in terms of field operations and earthquake characteristics - between this region and those used during the training phase. Nevertheless, this lends further support to the hypothesis that there are shared underlying patterns in the preparatory phases of the largest magnitude events in induced seismic sequences, which could be exploited through models able of complex non-linear analysis of the computed features.

**Figure 7:**
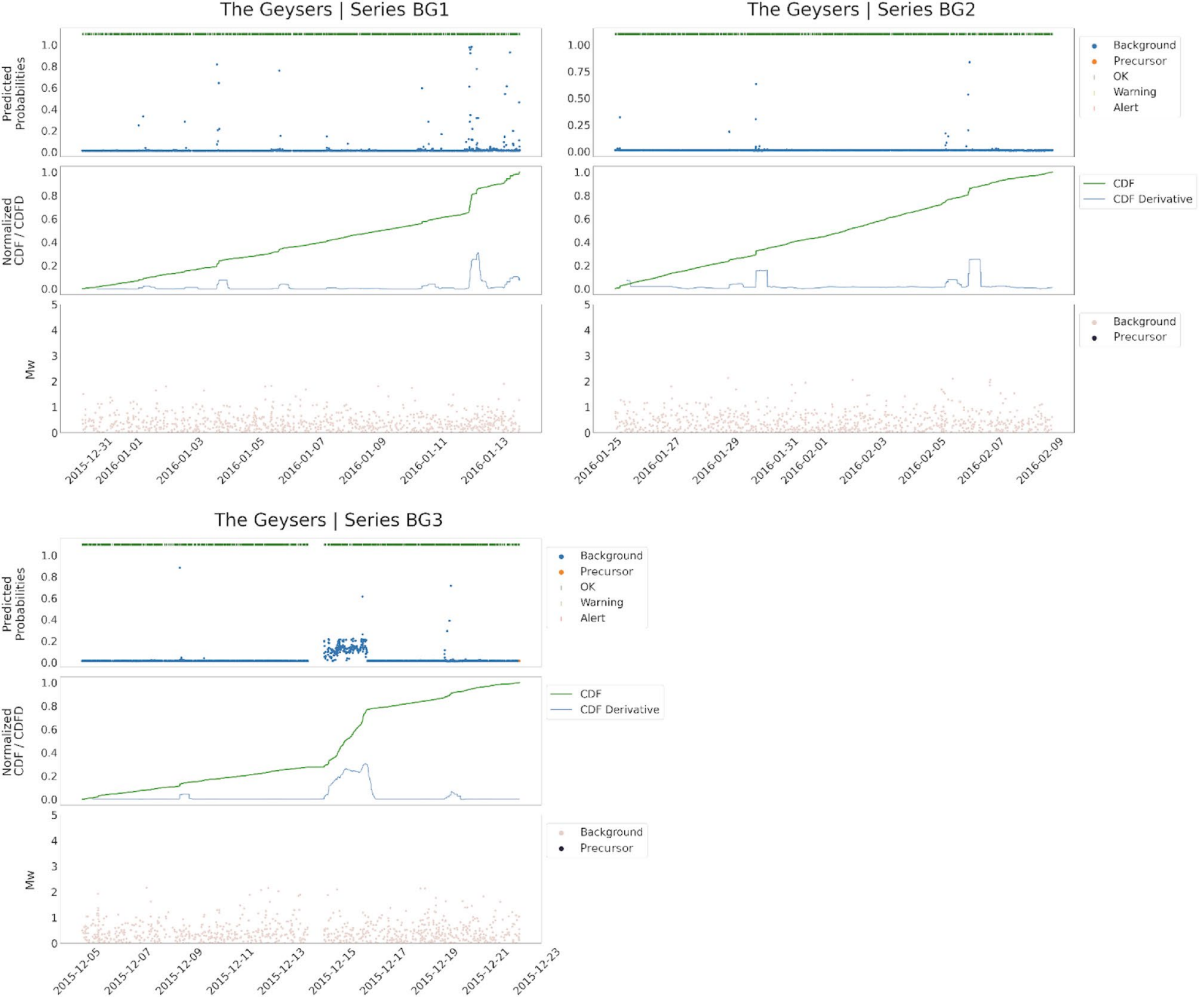
Test on background samples. The performances of the network have also been tested on sequences of events, extracted from the TG geothermal region, that do not contain any events of magnitude greater than 2.1. In this case, no precursors have been detected by the proposed methodology, and no warning is returned.

It should be underlined that the network produces a prediction in an element-wise manner, i.e. it returns the probability for a sample of belonging to the background class or to the precursor class: in this scenario, as long as a new event is detected, the features can be computed and the 16-dimensional vector associated can be passed through the classifier. In Fig. 7 a further experiment conducted with the aim of stressing the real-scenario applicability of the proposed workflow is shown. Three sequences of samples not containing any large earthquake have been extracted from the TG related catalogue, and the classification obtained by *PreD-Net* is reported. It can be observed how almost all the samples are correctly predicted as background seismicity, demonstrating that the network effectively recognizes patterns among the features that discriminate between a preparatory phase of a remarkable seismic event, in terms of magnitude, and background seismicity. Quantitatively, the number of samples exploited in the experiments seems to be sufficient to guarantee the convergence of the network. On the other hand, the differences in the results between the validation and the test sets prove that each collection of samples related to a specific large earthquake has, in a way, its patterns and peculiarities, also connected to the particular geothermal field to which they relate. In this sense, the biggest collection of time series, i.e. training contexts, could help the model to better generalise in a larger variety of environments.

Another important result concerns the fact that the approach has also worked by putting together data from earthquakes that have a different origin as a consequence of the different number of injection wells, their inter-distance^51,52^, and the amount, the rate and the pressure of the injected volume of fluids. In The Geyser geothermal field earthquakes mainly occur as a result of the injection of water or other fluids into hot rock, in Cooper Basin earthquakes originate from fracking operations. These latter are performed by injecting a mix of fluids (water, sand, and chemicals) at a pressure high enough to create new fractures or increase connectivity between fractures to allow oil and gas to escape from geological traps^53^. Additionally, the application to the Hengill geothermal field provides the first evidence that the proposed procedure can be potentially applied to natural earthquakes too. Indeed, it has been suggested that the Hengill dataset contains both induced and natural earthquakes^30^. Basel shares common features in terms of field operations with a part of The Geyers geothermal field, in particular with earthquakes that occurred during the Enhanced Geothermal System (EGS) Demonstration Project^54^

### Investigation on features’ importance

The findings derived from the implemented approach prompt an inquiry into the network’s ability to discern intricate patterns within the features set and identify the specific nature of these patterns. To rigorously evaluate the model’s sensitivity to the features set, a series of tests were designed to ascertain whether the *PreD-Net* model is capturing more than mere trivial relationships potentially observable in individual features. An examination of the features’ distribution reveals minimal discernible differences between background and precursor contexts. Consequently, statistical tests were employed to examine disparities in the mean, median, and variance between the distributions of features in the background and precursor contexts (as further elaborated in the section “Preliminary Investigation on Features’ importance and Validation of *PreD-Net* Results” of the Supplementary Materials).

Subsequent analysis confirms the presence of statistically significant differences among features within the two contexts, as evidenced by the results presented in Table S1. Nevertheless, the data do not reveal any explicit pattern that correlates a specific feature with a background or precursor label.

In an additional layer of investigation, this study also explores the comparative efficacy of simpler machine learning algorithms-such as Logistic Regression, Tree-based models, Support Vector Classification, and a rudimentary Multilayer Perceptron (MLP) -against the proposed *PreD-Net* model. This comparison was conducted using a 10-fold cross-validation procedure, accompanied by a feature importance analysis. The importance of individual features was evaluated using a permutation importance methodology. Specifically, during each iteration of the cross-validation process, one feature was randomly shuffled, and the resulting impact on model accuracy was recorded. Repeating this process ten times for each fold yields a robust estimate of each feature’s predictive power in relation to the applied model.

**Figure 8:**
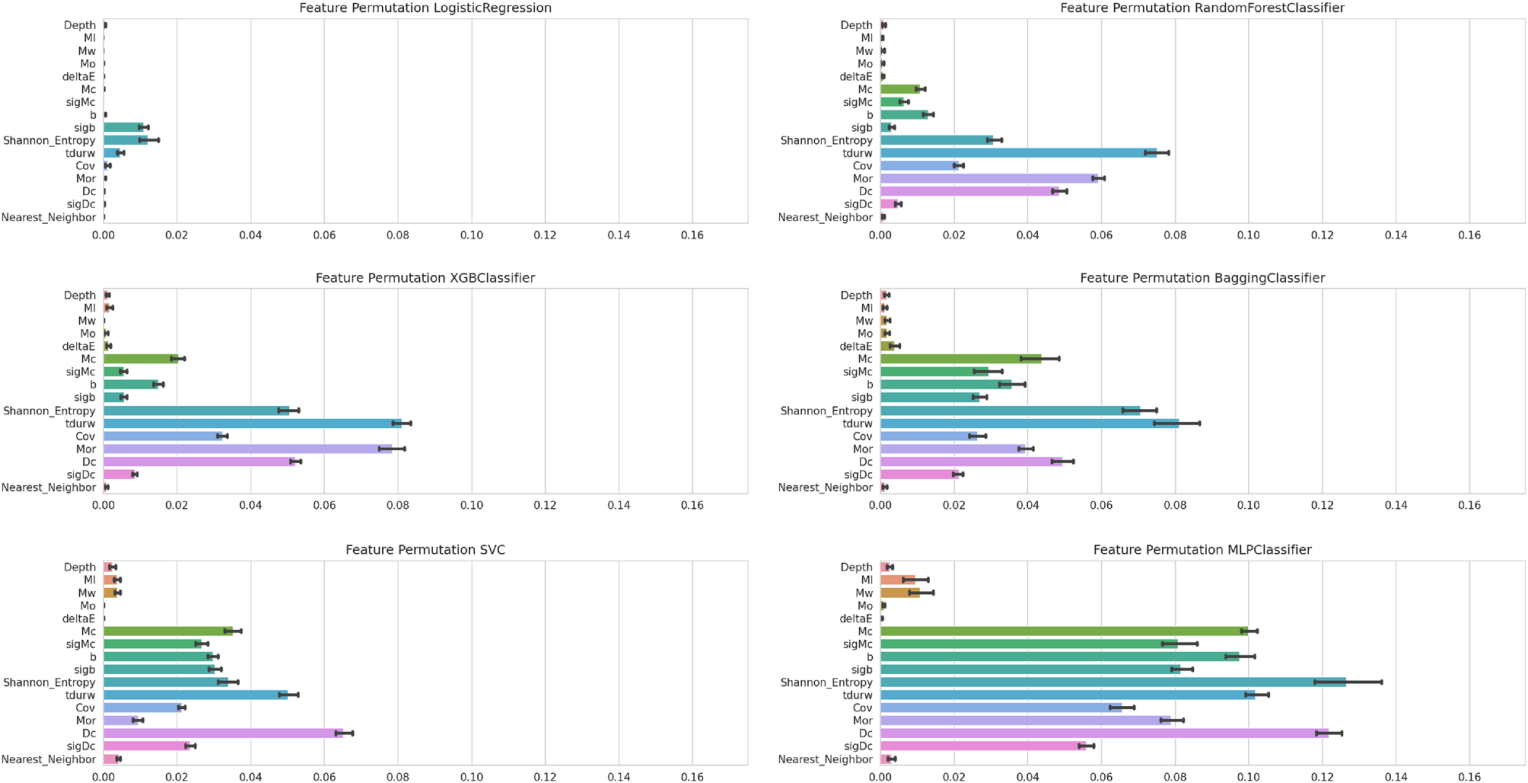
Feature importance. Feature importance, for machine learning algorithms, as determined through a features’ permutation process over a 10-fold cross validation.

As illustrated in Fig. 8, features that do not exhibit statistically significant variations between the two classes also display limited predictive utility, contributing minimally to the performance of all examined machine learning models. Conversely, no individual feature demonstrates a dominant influence, supporting the notion that no trivial patterns are identifiable for straightforward discrimination between precursor and background samples.

### Investigation on model performances

**Figure 9:**
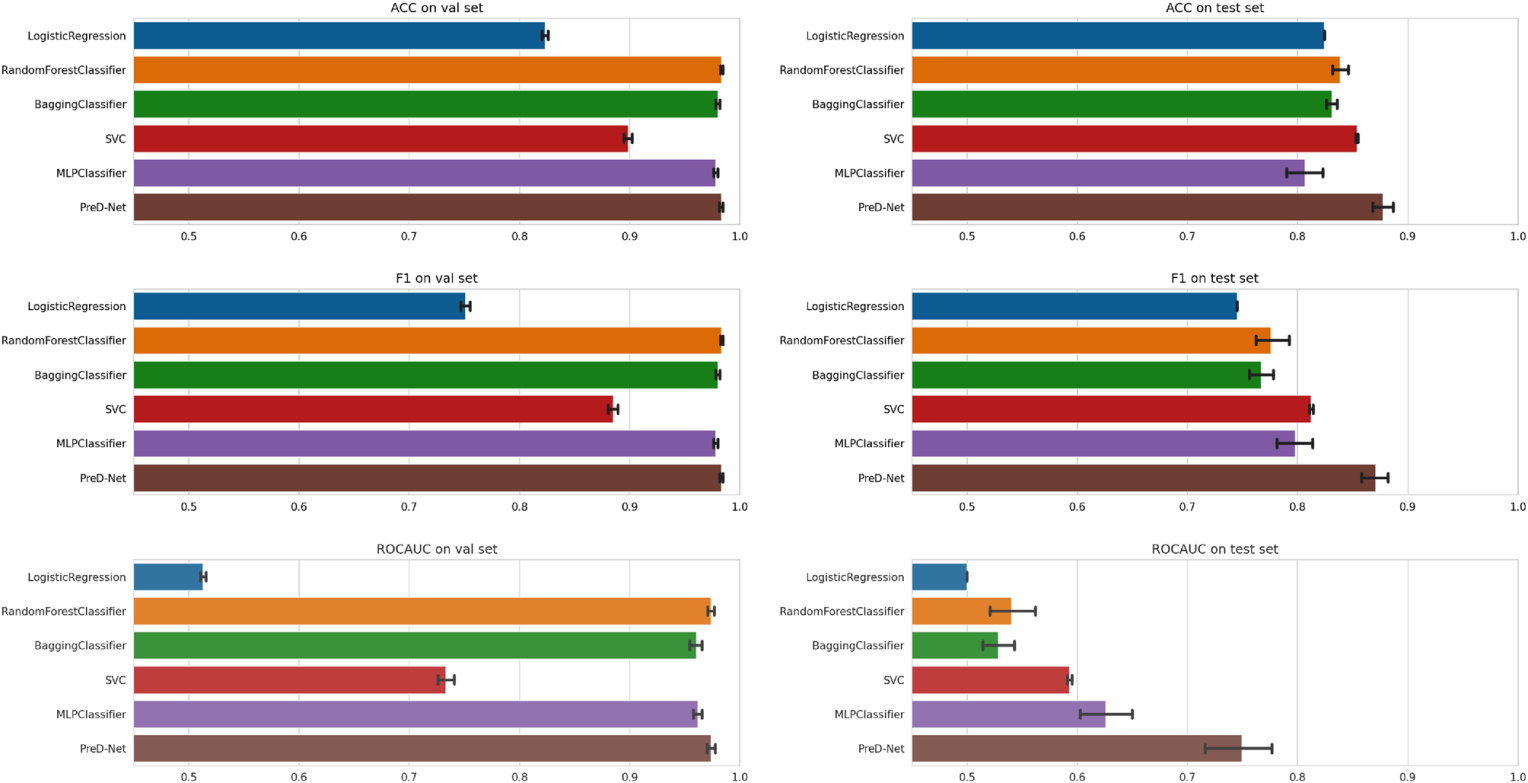
Comparative accuracy metrics. A side-by-side comparison of accuracy (ACC) metrics for Machine Learning algorithms and PreD-Net on both the validation and test sets.

The same methodology was employed to evaluate whether *PreD-Net* could effectively discern complex feature patterns that are not readily identifiable by other Machine Learning (ML) models. A performance assessment of these ML models was conducted using a 10-fold cross-validation procedure, the outcomes of which are illustrated in Fig. 9.

The suboptimal performance of rudimentary classifiers like logistic regression underscores the intricacy of the problem at hand. The skewed distribution of background samples relative to precursor samples adds another layer of complexity. Further insights can be drawn from the performance of tree-based algorithms and the Multilayer Perceptron. These algorithms, capable of capturing non-linear feature relationships, exhibit high levels of accuracy on the Validation set, demonstrating their ability to recognize known contexts with high confidence. However, they tend to fall short in terms of generalizability, particularly when faced with samples from previously unseen contexts (i.e. the *Test set*). In contrast, our proposed *PreD-Net* model excels not only in capturing patterns within known contexts but also in generalizing these patterns to predict unseen samples. As evidenced by Fig. 9, the model’s performance on the *Test set* significantly outpaces that of traditional ML algorithms. Elevated levels of accuracy, F1 score, and AUC score point to *PreD-Net*’s adeptness at distinguishing between precursor and background samples owing largely to its strong generalization capabilities.

### Timely alerts for induced seismicity

As for the possibility of real-time applications of the proposed technique, the results of the warning strategy suggest that a system based on three levels (No warning, Warning, and Alert) can be implemented, which can issue an alert hours before the occurrence of a significant earthquake in the framework of the induced seismicity. This may be of great help to avoid adverse consequences - social and economic - during field operations and reduce seismic hazard related to induced seismicity^55–58^. In fact, the computational times for the warning strategy are quite short, less than half a second to process three whole series, i.e. 4600 observations. Hence, they are negligible concerning all the other operations needed to collect and analyse data, i.e. earthquake location and magnitude estimation.

## Methods

Given the three original catalogues, the following statistical features have been computed: the minimum magnitude of completeness $Mc$, the $b$-value, moment magnitude ($M_W$), and moment rate, duration of events’ group, the coefficient of variation $CoV$, the Fractal Dimension, the Nearest-Neighbour distance, and the Shannon’s Information Entropy. All these quantities, except $M_W$ which is computed for each single event, are measured from sliding backward windows containing 200 events and overlapping by 1 event. As for the uncertainties that represent additional features used by the network, a bootstrap approach has been implemented.

Then, events of interest for the present study have been identified as events with larger moment magnitude in the considered catalogues. For the Geysers geothermal area, we selected the events with $M_W$ larger than 3.9, and for the Cooper Basin $M_W$ larger than 2.9. For the Hengill, events with a moment magnitude larger than 3.5 have been chosen. Around the selected events, 2000 consequential samples have been extracted, where the event of interest corresponds to the sample 1501, in the case of the first two geothermal fields; for the third one, 600 events around the largest event have been considered, where the highest moment magnitude event is located at the 351st sample (see Supplementary Materials for further details).

This collection of samples represents the dataset under analysis, whose elements have been labelled according to the procedure discussed in the following sections. It is worth noting that the dataset turns out to be strongly unbalanced since, for each large earthquake related series of events, the precursors samples are usually limited to a zone close to the large event itself and end just before it, while the remaining part of the samples is marked as background seismicity. This results in a disproportion between the two classes to be recognized, turning the classification task into a rare event recognition problem, which, however, reflects the seismological reality. Moreover, artificially reducing the number of background points to balance the dataset can lead to the erroneous recognition of “background regions” in the features’ space, degrading the model’s performance. Hence, no rebalancing procedure on the dataset has been applied.

To maintain the coherence between the information collected from different sources, data have been normalized in the interval $[-1, 1]$ separately for samples collected in the three regions. This decision is twofold: on one side, normalization is essential to ensure a fair comparison between quantities collected in areas where seismic events have different origins; on the other side, fitting different scalers on data from various regions ensures their applicability to new samples from known areas.

### Statistical parameters

In the present section, we specify the statistical parameters used as input to the network to discriminate the potentail precursors. Before computing any parameter, we convert the local magnitude $M_L$ into moment magnitude $M_W$. For the Geysers, we used the relationship calibrated by^59^. For Cooper Basin, we estimated the relation using the data for the Habanero 4 well reported on the IS-EPOS platform, while for Hengill, we used the relation proposed by^60^. As for Basel, the used catalogue does already contain the moment magnitude.

Next, we compute the following parameters: the minimum magnitude of completeness ($Mc$), the $b$-value of the Gutenberg-Richter relation, the moment rate, the total duration of selected groups of events, the coefficient of variation ($CoV$), the mean value of the inter-event times, fractal dimension ($Dc$), Nearest-Neighbour Distance^61^ ($\eta$), and the Shannon entropy^62^ ($H$).

As for $Mc$, which is critical for reliable estimates of the $b$-value, we use the maximum curvature technique^63^. The $b$-value is estimated by applying the maximum likelihood approach^64^. The Moment rate, the total duration of event groups, and the coefficient of variation, that is, the ratio between the standard deviation and the mean value of the inter-event times, were computed for each sliding window.

The fractal dimension $D_{c}$ was estimated using the formula $D_{c} = \lim _{r \rightarrow 0}[\log C(r)/log r]$, where $r$ is the radius of the research sphere, and $C(r)$ is the correlation integral evaluated on the number of window points^65^.

The Nearest-Neighbour Distance, which represents a particular distance between events that combine space, time, and magnitude information^61^, is obtained as $\log \eta = \log R_{ij} + \log T_{ij}$, where $T_{ij}$ and $R_{ij}$ are the rescaled distance and time^61^. The Shannon entropy $H$ represents a measure of the disorder level in a system; it has been computed as $H = -\sum _{k=1}^{n} P_{k}(E)[\ln {P_{k}(E)}]$, where $P_{k}(E)$ is the probability that a fraction of the total seismic energy is radiated within the $k^{th}$ cell^66^. The total seismic energy has been computed using the relation proposed for the earthquakes in California^67^.

### Precursors labelling

The subsequent step consists of the identification in each sequence of events related to a large event of potential precursors with respect to the background seismicity and subsequent events in the sequence. To this end, for each identified main event, we compute the associated source radius assuming a circular source rupture model^68^ ($r = \left( \frac{7}{16}\frac{M_{o}}{\Delta \sigma }\right) ^{1/3}$) and an average stress drop value $\Delta \sigma = 1$ MPa valid for the Geysers area^59^ and $\Delta \sigma = 20$ MPa for the Hengill area^69^. Next, we assume that an event can be classified as a potential precursor if it is located in an area with a radius twice that of the one obtained from the source model and occurs before the principal event in a specific time window. The duration of the time window is selected by analysing the $\beta$-statistic, which is used to compare the seismicity rate of two periods^70^, and the cumulative seismic moment. In particular, in a time window of 35% of the total duration of the time-series, the jumps in the cumulative moment are selected as candidates for the start of the preparatory phase. The coherence of this choice is then evaluated through the $\beta$-statistic. The results suggest that this choice is suitable in most cases. However, none of the investigated parameters can be used alone without user support, and the final choice for all the series has been conducted according to domain expertise. The final duration ranges between 20 hours and 20 days. This duration is coherent with that used to forecast labquakes by using Deep Learning^48^.

More specifically, in the present study, for consecutive time intervals $(t_i, t_{i+1})$ we compare the corresponding seismicity rates $r_i$ and $r_{i+1}$, and compute when the two rates are statistically different, which is identified by positive values of the $\beta$ statistic, i.e. increasing seismicity. When this condition is verified, we start to label the earthquakes as candidate precursors up to the occurrence of the large event. The $\beta$ statistic is thus defined as:

1 $$\begin{aligned} \beta (n_{i+1},n_{i}, t_{i+1}, t_{i})=\frac{n_{i+1}-E(n_i+1)}{\sqrt{var{(n_{i+1})}}} \end{aligned}$$

where $n_{i+1} = t_{i+1} \cdot r_{i+1}$ and $n_i = t_i \cdot r_i$. $E(n_{i+1}) = r_{i} \cdot t_{i+1}$ is the value of $n_{i+1}$ expected under the null hypothesis that the earthquake occurrence has a distribution similar to that observed in the time interval $t_i$. The symbol *var* denotes variance and for the present application, assuming a Poisson process, corresponds to $r_{i} \cdot t_{i+1}$, that is, to the $E(n_{i+1}) = r_{i} \cdot t_{i+1}$. The analysis of the obtained results suggests a proper duration for the time window to be 35% of the total duration of the time series for each mainshock. In practice, the durations range between 20 hours and 20 days.

### PreD-net architecture

**Figure 10:**
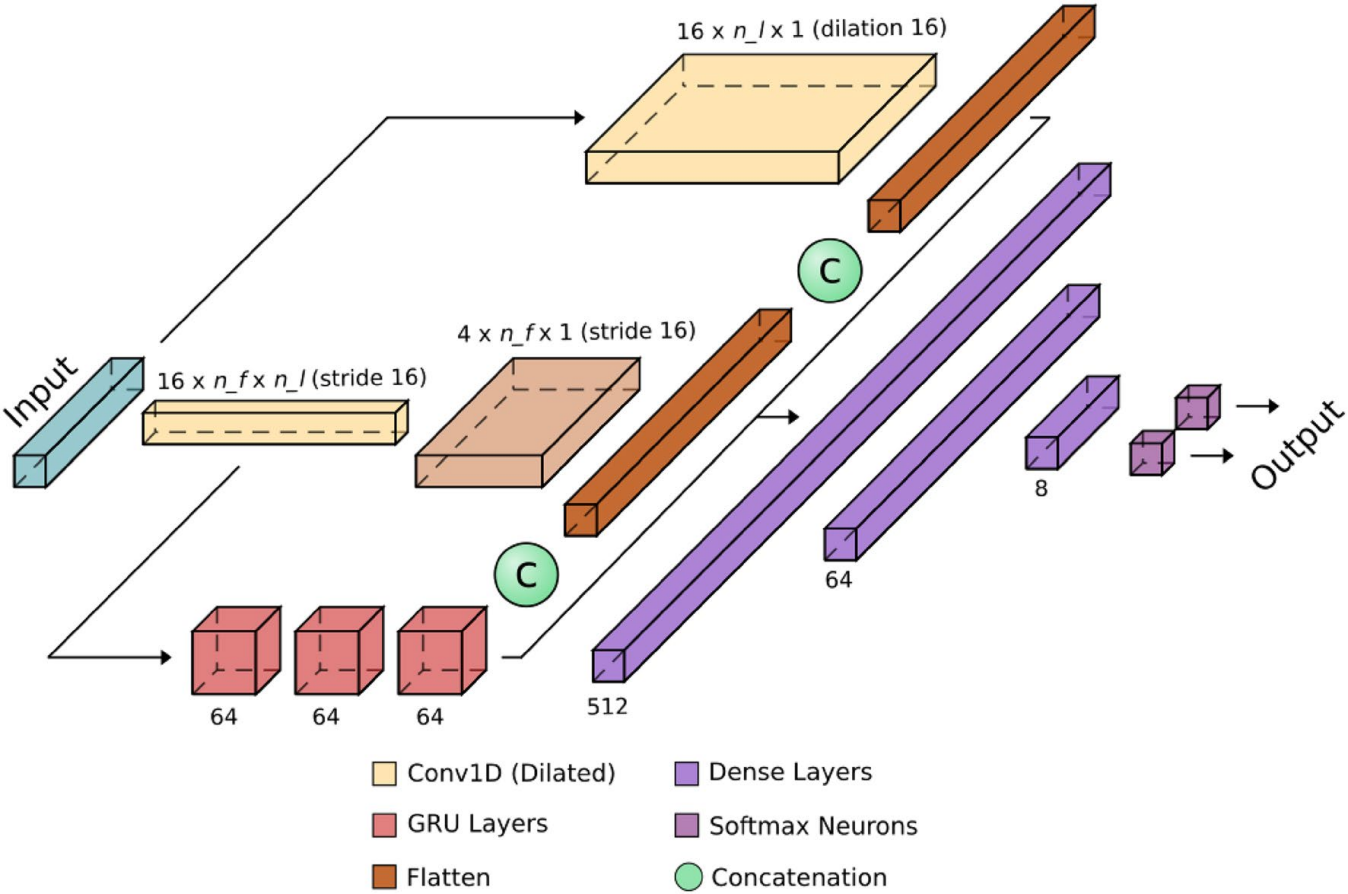
The PreD-Net architecture. The PreD-Net used for the prediction of precursors, is designed to operate with and without lagged variables. The central branch acts as a convolutional autoencoder on the feature of the problem, generating a latent space of dimension $n_lx16$, where $n_l$ is the number of lagged variables. The GRU layers act on the latent space reconstructing the temporal dependencies between the $n_l$ lagged considered steps. At the same time, a dilated convolution extracts the relations between every single feature with its lagged versions. The three outputs are concatenated and fed to a dense sequence of layers to ensemble the extracted information. A softmax output returns the probability of each sample belonging to class 0 (background seismicity) or class 1 (precursors).

The model adopted for the classification of the precursors is a three-branch neural network, as reported in Fig. 10. This network has been designed to operate with or without lagged variables (see Supplementary Materials): the central branch of the network, constituted by 1D convolutional and transposed convolutional filters, acts as an Autoencoder, extracting relations between the different features of each sample and projecting them into a latent space. This latent space is used as input as a second branch made up of recurrent layers, in particular GRU ones, which has the task of analysing the potential time relation between lagged versions of the sample under consideration, i.e. to take into account the temporal patterns. The third branch, built upon dilated 1D convolution, aims to investigate relations between lags of each feature so that their time self-dependencies are considered. The three branches are then concatenated, and their output is fed through dense layers to a softmax output, which returns the probability of belonging to each of the two classes of the problem, namely foreshocks or background seismicity.

### Classification metrics

To evaluate the performances obtained by the *PreD-Net* in the classification task, we exploit four of the most common metrics for classification problems^71^. The first one is Accuracy, which is the percentage of correct predicted observations. Then, there are Precision and Recall, which represent, respectively, the percentage of correctly predicted precursors on the total predicted precursors and the ratio of correctly predicted precursors on their total. Finally, the F1 score takes into account both Precision and Recall by considering their harmonic mean. In formula:

2 $$\begin{aligned} Pr = \frac{TP}{TP + FP}; \qquad Rec = \frac{TP}{TP + FN}; \qquad F1 = 2\frac{Pr \cdot Rec}{Pr + Rec} \end{aligned}$$

where TP, FP and FN are, respectively, the True Positive, False Positive and False Negative, i.e. the number of precursors correctly predicted, the number of precursors wrongly predicted as background motions and the number of background motions wrongly predicted as precursors. In order to provide reliable measurements of the actual performances of the PreD-Net model, all the aforementioned metrics, except the accuracy, are calculated for each label and weighted with respect to their support.

### The warning strategy

**Figure 11:**
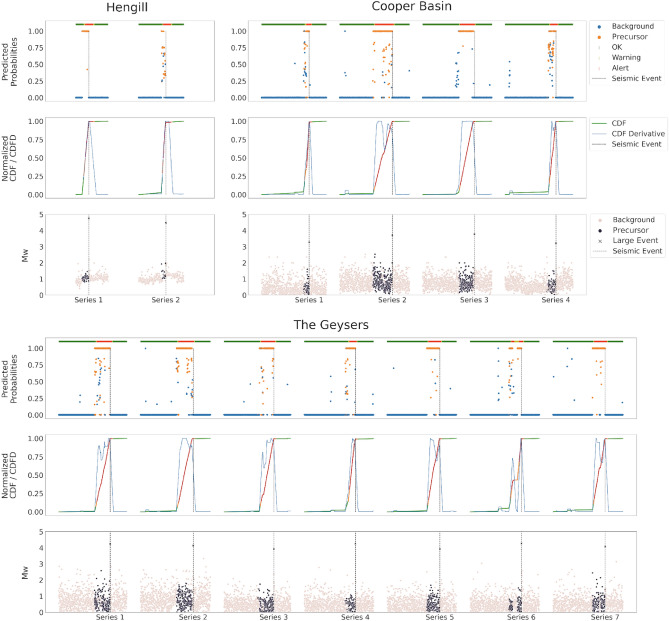
The results of PreD-Net in the validation set. The upper-left sub-figure refers to the events of the Hengill geothermal field, the upper-right one to samples of the Cooper basin. In the lower sub-figure, the series of The Geysers geothermal field are reported. In particular, for each figure, the upper panel reports the probability a certain earthquake is labelled as a “Precursor” by the PreD-Net (value on y-axis), while the colour indicates the ground truth (blue points correspond to the background samples, orange ones to the precursors). The upper bar represents the three levels of warning: green for no warning, orange for forthcoming alarm and red for a strong alert. The dotted vertical line represents the largest event position in the time-series. In the middle panel, the CDF is represented by the green-orange-red line (also, in this case, the colours follow the warning system). The blue line represents the CDFD, which is the one that affects the early warning. The bottom panel contains the magnitude ($M_W$) of the events, divided into Background (pink points) and Precursors ( dark points).

**Figure 12:**
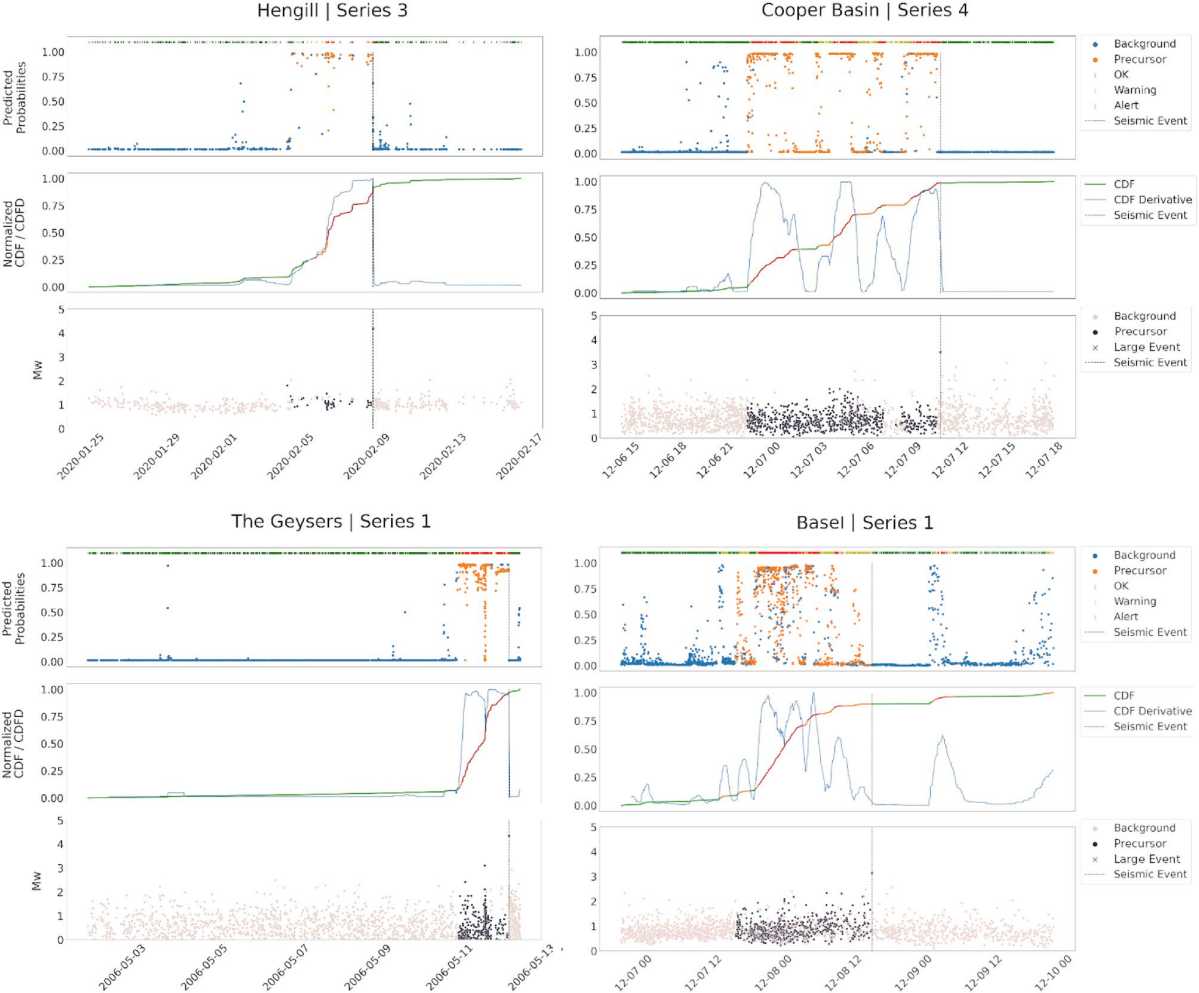
The results of classification and warning for the test series. The figure follows the scheme proposed in Fig. 11. In particular, in the middle panel, can be observed how the warning system is triggered as PreD-Net starts recognizing precursor windows. On the x-axis of the plots, there is the time at which the events occur.

A warning strategy has been designed according to classification results on the validation set and the precursors’ identification of completely unknown series related to seismic events of interest. In particular, the strategy mentioned above is based on the cumulative predicted probability of belonging to the class “precursors” and on its slope. Since the model shows high accuracy in predicting the background seismicity, especially in the region preceding the largest events, for each series, the cumulative probability of a “precursor” classified sample is computed. When the increment of such a cumulative distribution shows a steep slope, i.e. the number of predicted “precursors” among samples is high and contiguous, a warning is activated (represented by orange zones of the alert in Figs. 11, 12). If the slope further increases, the warning turns into a red alert, reporting a high risk of being in a real “precursor” zone that anticipates a considerable magnitude event. More specifically, two thresholds are fixed. When the CDFD exceeds the lower threshold, then the warning is activated, when it is also above the upper threshold, then a red alert is given. Furthermore, there is also another condition. If the CDFD decreases from above the upper threshold to below the lower one and the CDF is at a high value, then an orange signal is given instead of a green one. This condition is set as it is reliable that there is still a dangerous situation, as for the Cooper Basin test series in Fig. 12. Finally, the alarm stops if there are no further signals in the next 24 hours.

### Supplementary Information

Supplementary Information.

## Data Availability

The data used in this study for the Geysers and Cooper Basin geothermal fields are available at https://doi.org/10.5281/zenodo.7733489 and https://episodesplatform.eu/?lang=en#datasearch:episode=COOPER_BASIN &dataType=Catalog, respectively. Basel catalog is available as electronic supplement from: Herrmann, M., T. Kraft, T. Tormann, L. Scarabello, and S. Wiemer. (2019). “A Consistent High-resolution Catalog of Induced Seismicity in Basel Based on Matched Filter Detection and Tailored Post-processing” Journal of Geophysical Research: Solid Earth 124, doi:10.1029/2019JB017468.
