# Author Correction: Deep learning forecasting of large induced earthquakes via precursory signals

**DOI:** 10.1038/s41598-024-59628-w

**Published:** 2024-04-22

**Authors:** Vincenzo Convertito, Fabio Giampaolo, Ortensia Amoroso, Francesco Piccialli

**Affiliations:** 1 Istituto Nazionale di Geofisica e Vulcanologia, Osservatorio Vesuviano, Naples, Italy; 2 Department of Mathematics and Applications “R. Caccioppoli”, Univeristy of Naples Federico II, Naples, Italy; 3 Department of Physics “E.R. Caianiello”, University of Salerno, Fisciano, SA Italy

Correction to: *Scientific Reports* 10.1038/s41598-024-52935-2, published online 05 February 2024

The original version of this Article contained errors.

In the original version of this article, the legend of Table 1 was omitted. It now reads,

“Metric results about PreD-Net.”

Furthermore, the Acknowledgements section contained an error in one of the project numbers.

“The publication was produced with the co-financing of the European Union - Next Generation EU. This publication has been supported by the project titled D.I.R.E.C.T.I.O.N.S. - Deep learning aIded foReshock deteCTIOn Of iNduced mainShocks, project code: P20220KB4F, CUP: E53D23032910001, PRIN 2022 - Piano Nazionale di Ripresa e Resilienza (PNRR), Mission 4 “Istruzione e Ricerca” - Componente C2 Investimento 1.1, “Fondo per il Programma Nazionale di Ricerca e Progetti di Rilevante Interesse Nazionale (PRIN)”. The figures are generated by using the version 5 of Generic Mapping Tools (https://www.generic-mapping-tools.org/) [GMT 5: Wessel, P., W. H. F. Smith, R. Scharroo, J. Luis, and F. Wobbe, Generic Mapping Tools: Improved Version Released, EOS Trans. AGU, 94(45), p. 409-410, 2013. doi:10.1002/2013EO450001].”

now reads,

“The publication was produced with the co-financing of the European Union - Next Generation EU. This publication has been supported by the project titled D.I.R.E.C.T.I.O.N.S. - Deep learning aIded foReshock deteCTIOn Of iNduced mainShocks, project code: P20220KB4F, CUP: E53D23021910001, PRIN 2022 - Piano Nazionale di Ripresa e Resilienza (PNRR), Mission 4 “Istruzione e Ricerca” - Componente C2 Investimento 1.1, “Fondo per il Programma Nazionale di Ricerca e Progetti di Rilevante Interesse Nazionale (PRIN)”. The figures are generated by using the version 5 of Generic Mapping Tools (https://www.generic-mapping-tools.org/) [GMT 5: Wessel, P., W. H. F. Smith, R. Scharroo, J. Luis, and F. Wobbe, Generic Mapping Tools: Improved Version Released, EOS Trans. AGU, 94(45), p. 409-410, 2013. doi:10.1002/2013EO450001].”

The original Article has been corrected.

