# Supplementary material for: Deep learning forecasting of large induced earthquakes via precursory signals
Source: Sci Rep. 2024 Feb 5;14:2964. doi: 10.1038/s41598-024-52935-2 (PMC10844195; doi:10.1038/s41598-024-52935-2)
Supplement: Supplementary file 1 — Supplementary Information. [file 41598_2024_52935_MOESM1_ESM.pdf]

Deep learning forecasting of large induced earthquakes via precursory signals

Vincenzo Convertito[1], Fabio Giampaolo[2], Ortensia Amoroso[3], and Francesco Piccialli[2,*]

[1]Istituto Nazionale di Geofisica e Vulcanologia, Osservatorio Vesuviano, Napoli, Italy
[2]Department of Mathematics and Applications ”R. Caccioppoli”, Univeristy of Naples Federico II, Italy
[3]Department of Physics “E.R. Caianiello”, University of Salerno, Fisciano (SA), Italy
[*]francesco.piccialli@unina.it

ABSTRACT

Features of the Extracted Time Series

In the following, the plot of normalized computed features for the time series investigated in the manuscript is reported.

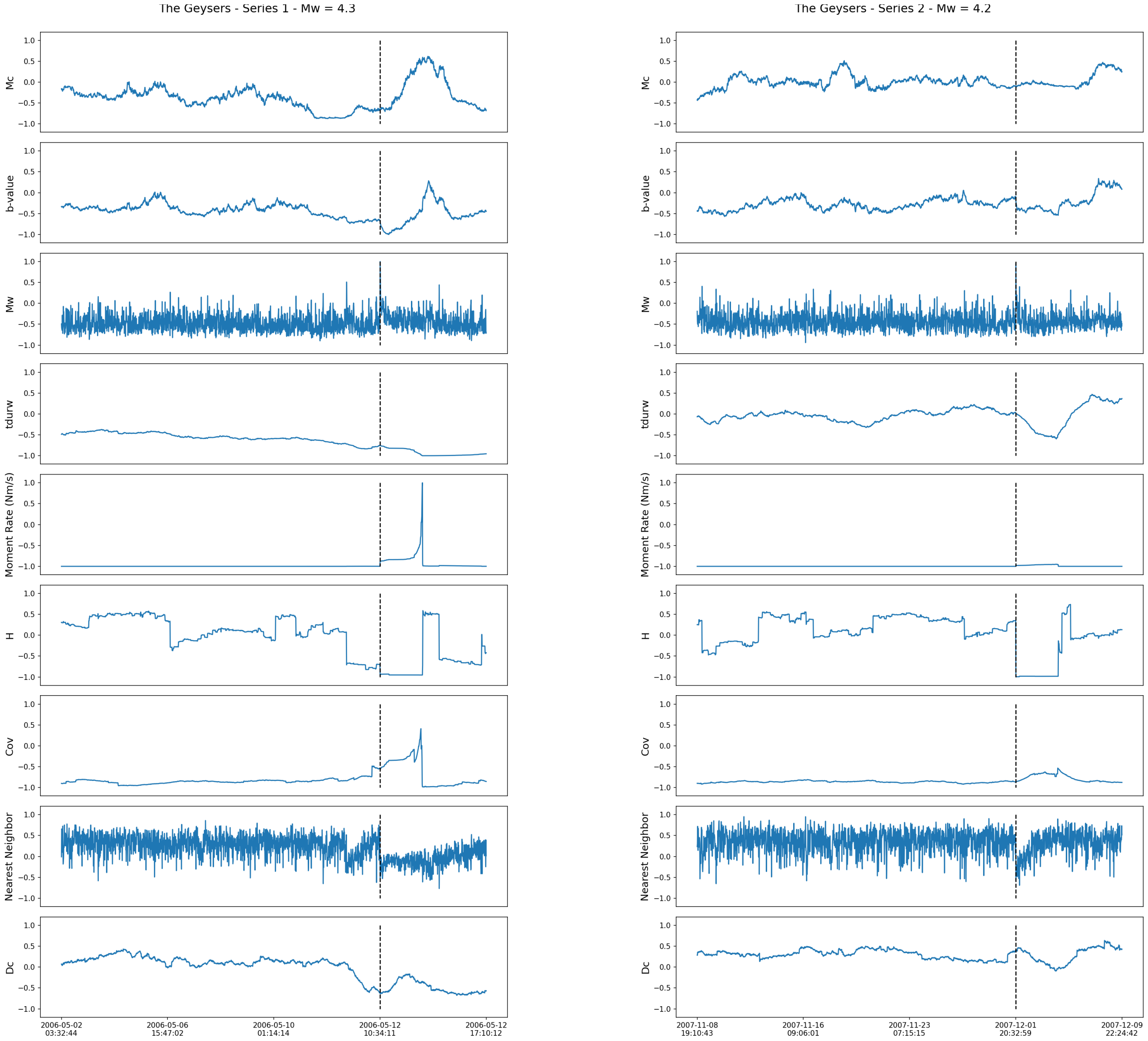
The Geysers - Series 1 - Mw = 4.3
The Geysers - Series 2 - Mw = 4.2
Mc
b-value
Mw
tdurw
Moment Rate (Nm/s)
H
Cov
Nearest Neighbor
Dc
2006-05-02 03:32:44
2006-05-06 15:47:02
2006-05-10 01:14:14
2006-05-12 10:34:11
2006-05-12 17:10:12
2007-11-08 19:10:43
2007-11-16 09:06:01
2007-11-23 07:15:15
2007-12-01 20:32:59
2007-12-09 22:24:42

(a) **(b)**

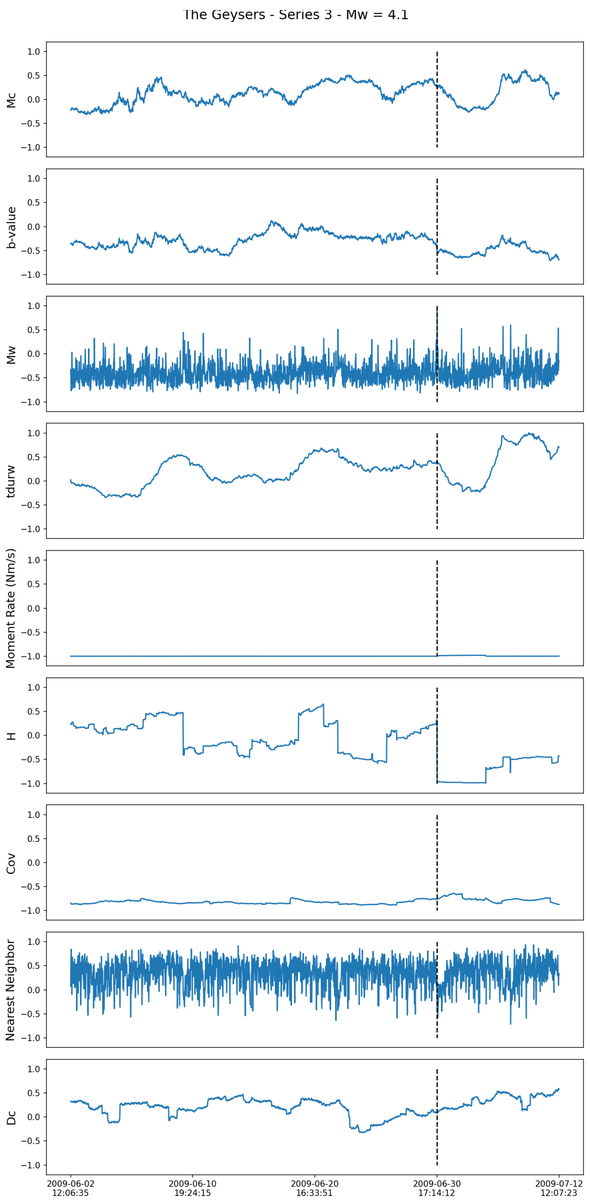
The Geysers - Series 3 - Mw = 4.1
Mc
b-value
Mw
tdurw
Moment Rate (Nm/s)
H
Cov
Nearest Neighbor
Dc
2009-06-02 12:06:35
2009-06-10 19:24:15
2009-06-20 16:33:51
2009-06-30 17:14:12
2009-07-12 12:07:23

(c)

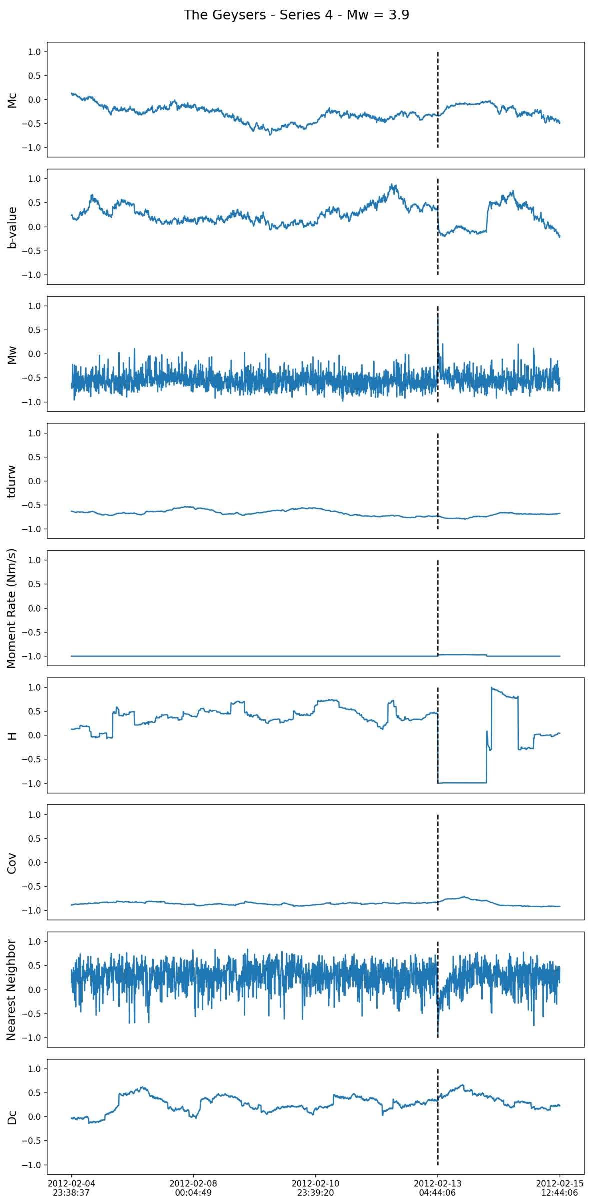
The Geysers - Series 4 - Mw = 3.9
Mc
b-value
Mw
tdurw
Moment Rate (Nm/s)
H
Cov
Nearest Neighbor
Dc
2012-02-04 23:38:37
2012-02-08 00:04:49
2012-02-10 23:39:20
2012-02-13 04:44:06
2012-02-15 12:44:06

(d)

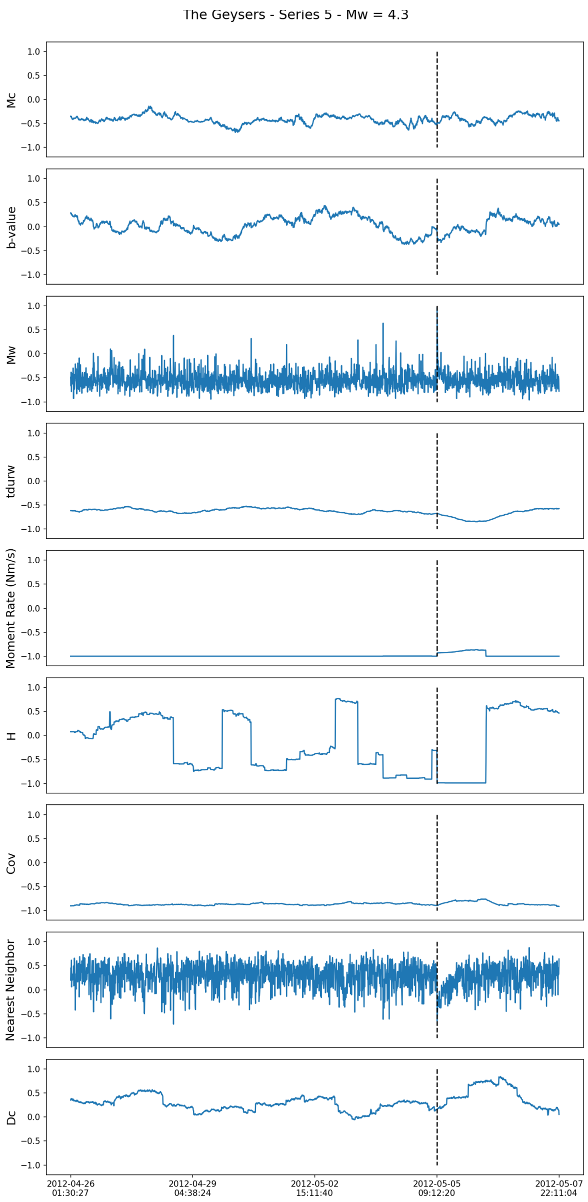
The Geysers - Series 5 - Mw = 4.3
Mc
b-value
Mw
tdurw
Moment Rate (Nm/s)
H
Cov
Nearest Neighbor
Dc
1.0
0.5
0.0
−0.5
−1.0
2012-04-26 01:30:27
2012-04-29 04:38:24
2012-05-02 15:11:40
2012-05-05 09:12:20
2012-05-07 22:11:04

(e)

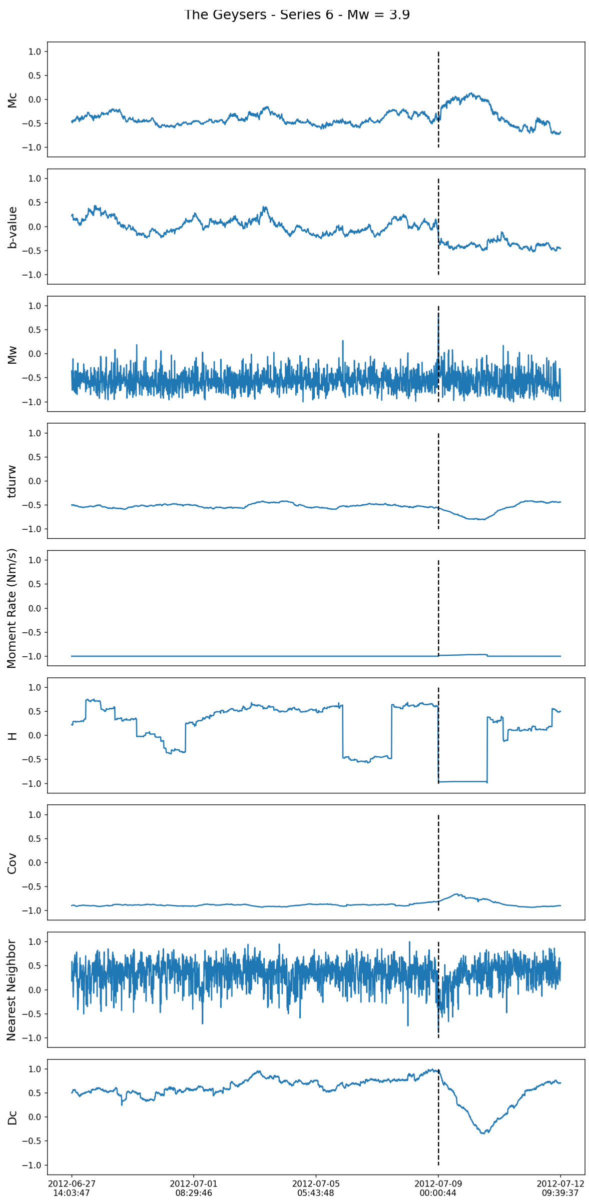
The Geysers - Series 6 - Mw = 3.9
Mc
b-value
Mw
tdurw
Moment Rate (Nm/s)
H
Cov
Nearest Neighbor
Dc
1.0
0.5
0.0
−0.5
−1.0
2012-06-27 14:03:47
2012-07-01 08:29:46
2012-07-05 05:43:48
2012-07-09 00:00:44
2012-07-12 09:39:37

(f)

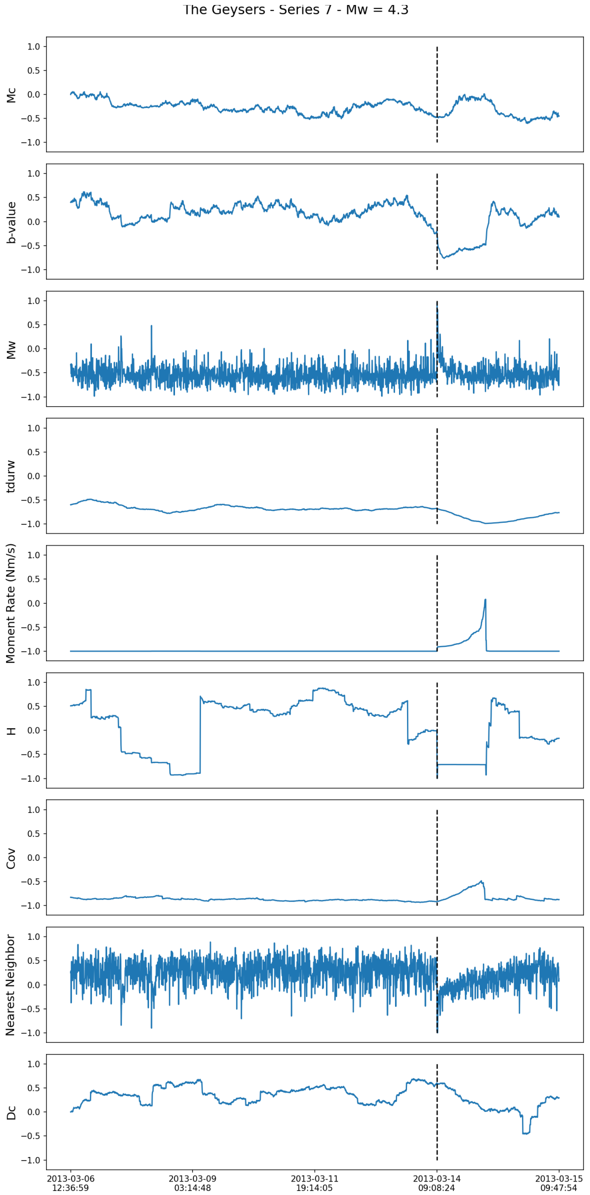
The Geysers - Series 7 - Mw = 4.3
Mc
b-value
Mw
tdurw
Moment Rate (Nm/s)
H
Cov
Nearest Neighbor
Dc
2013-03-06 12:36:59
2013-03-09 03:14:48
2013-03-11 19:14:05
2013-03-14 09:08:24
2013-03-15 09:47:54

(g)

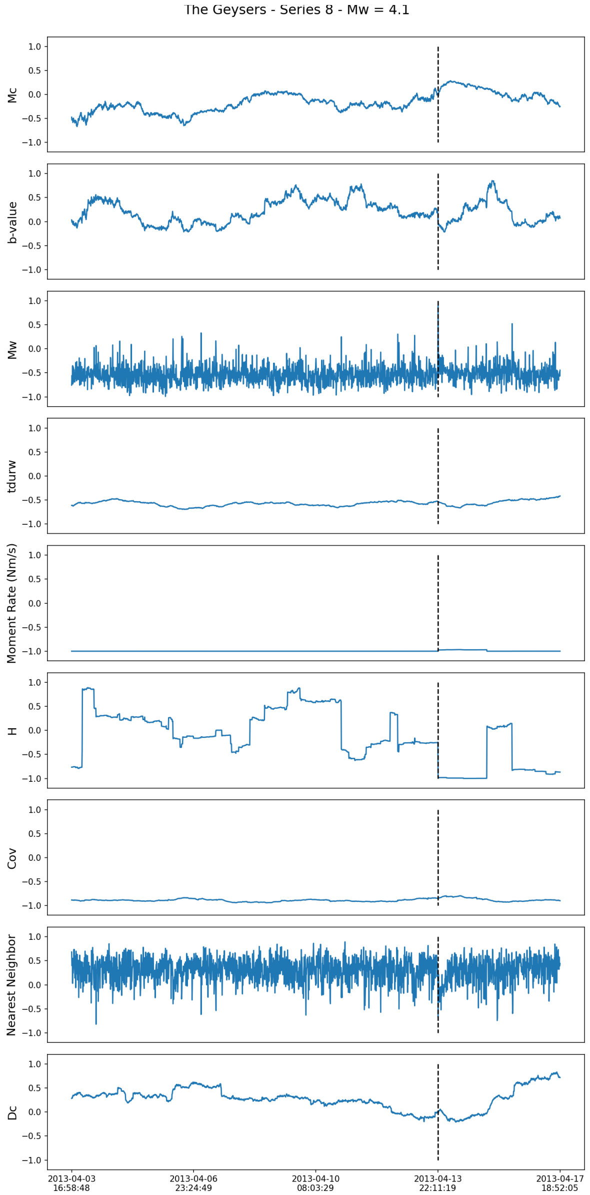
The Geysers - Series 8 - Mw = 4.1
Mc
b-value
Mw
tdurw
Moment Rate (Nm/s)
H
Cov
Nearest Neighbor
Dc
2013-04-03 16:58:48
2013-04-06 23:24:49
2013-04-10 08:03:29
2013-04-13 22:11:19
2013-04-17 18:52:05

(h)

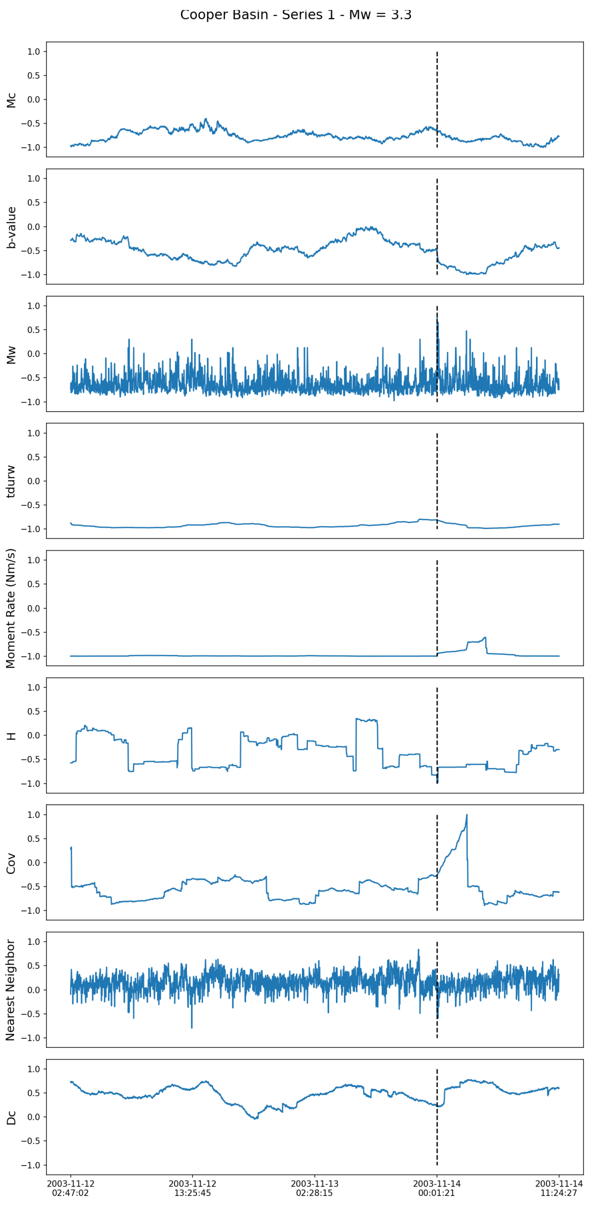
Cooper Basin - Series 1 - Mw = 3.3
Mc
b-value
Mw
tdurw
Moment Rate (Nm/s)
H
Cov
Nearest Neighbor
Dc
2003-11-12 02:47:02
2003-11-12 13:25:45
2003-11-13 02:28:15
2003-11-14 00:01:21
2003-11-14 11:24:27

(i)

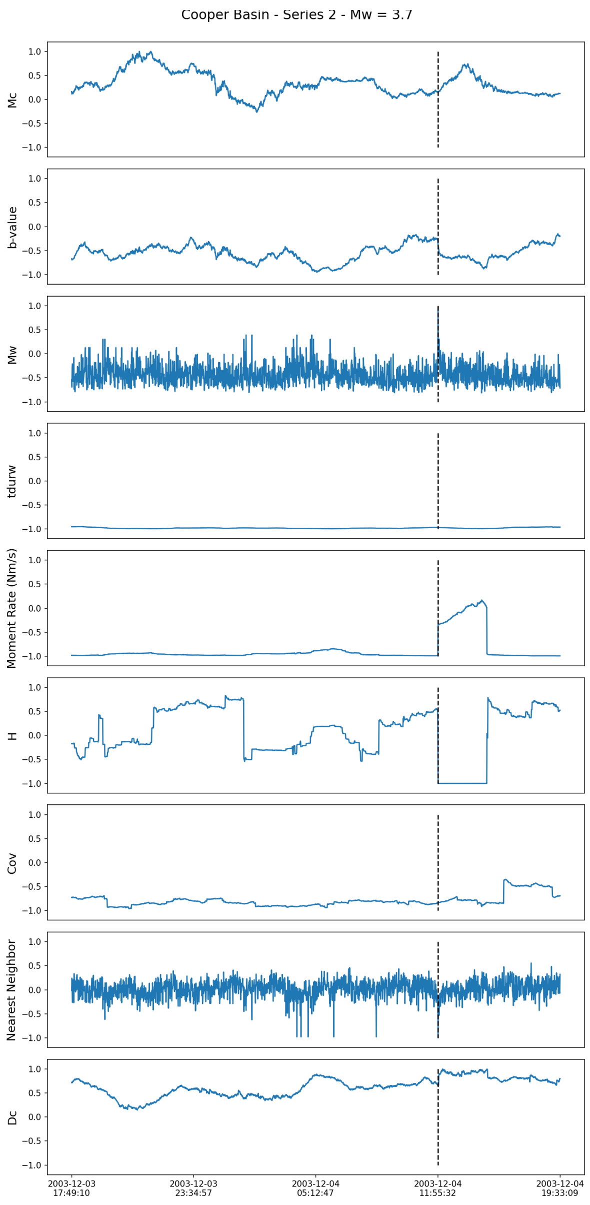
Cooper Basin - Series 2 - Mw = 3.7
Mc
b-value
Mw
tdurw
Moment Rate (Nm/s)
H
Cov
Nearest Neighbor
Dc
2003-12-03 17:49:10
2003-12-03 23:34:57
2003-12-04 05:12:47
2003-12-04 11:55:32
2003-12-04 19:33:09

(j)

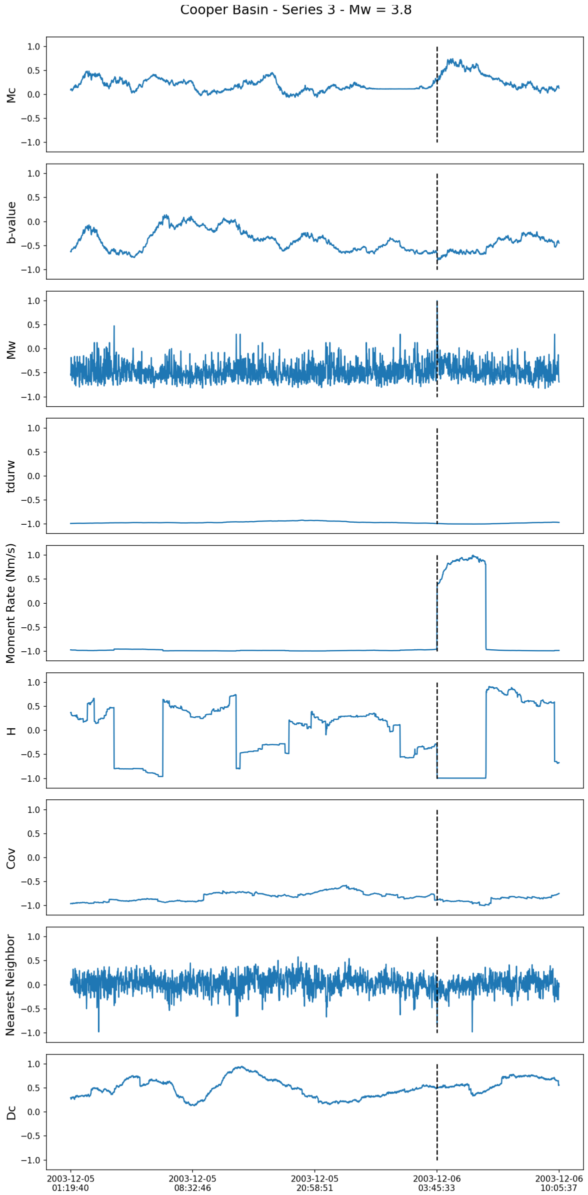
Cooper Basin - Series 3 - Mw = 3.8
Mc
b-value
Mw
tdurw
Moment Rate (Nm/s)
H
Cov
Nearest Neighbor
Dc
2003-12-05 01:19:40
2003-12-05 08:32:46
2003-12-05 20:58:51
2003-12-06 03:45:33
2003-12-06 10:05:37

(k)

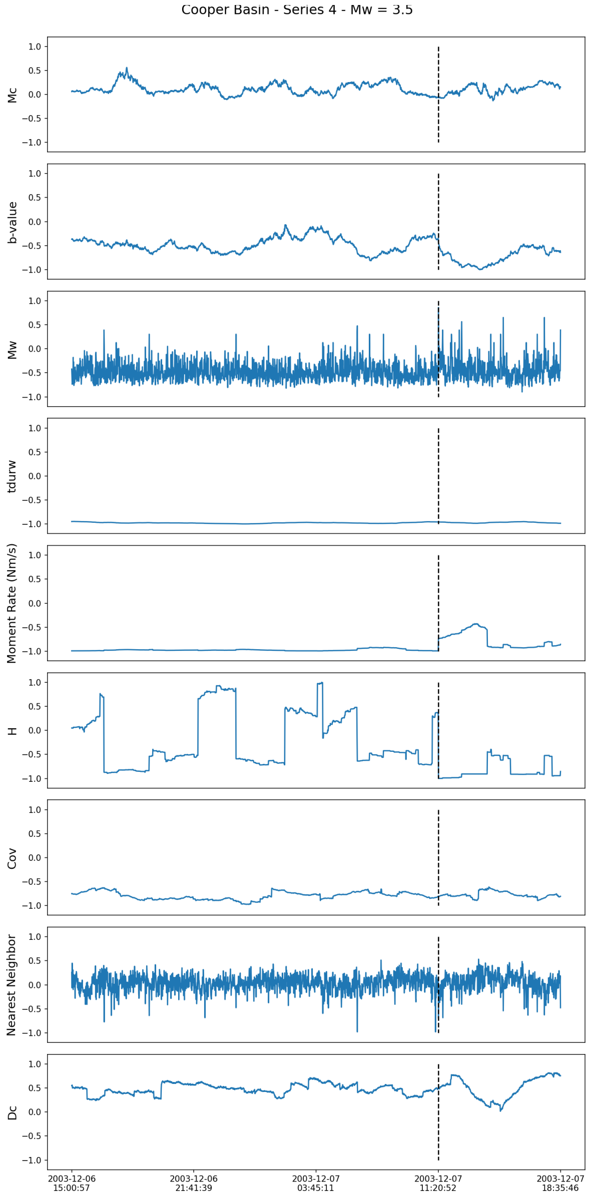
Cooper Basin - Series 4 - Mw = 3.5
Mc
b-value
Mw
tdurw
Moment Rate (Nm/s)
H
Cov
Nearest Neighbor
Dc
2003-12-06 15:00:57
2003-12-06 21:41:39
2003-12-07 03:45:11
2003-12-07 11:20:52
2003-12-07 18:35:46

(l)

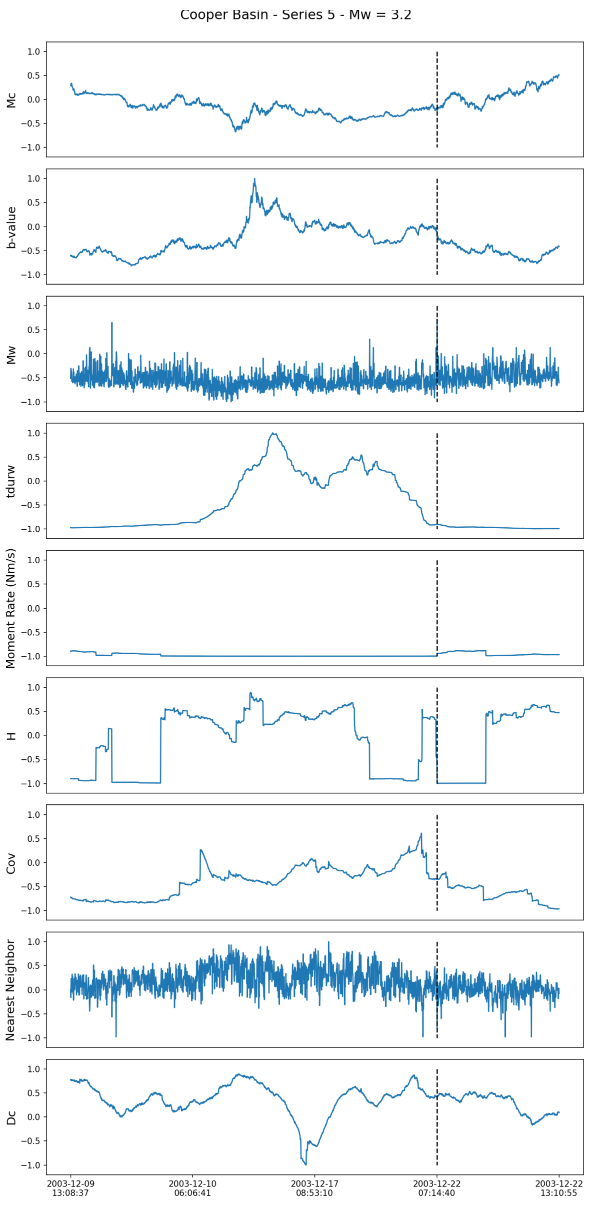
Cooper Basin - Series 5 - Mw = 3.2
Mc
b-value
Mw
tdurw
Moment Rate (Nm/s)
H
Cov
Nearest Neighbor
Dc
2003-12-09 13:08:37
2003-12-10 06:06:41
2003-12-17 08:53:10
2003-12-22 07:14:40
2003-12-22 13:10:55

(m)

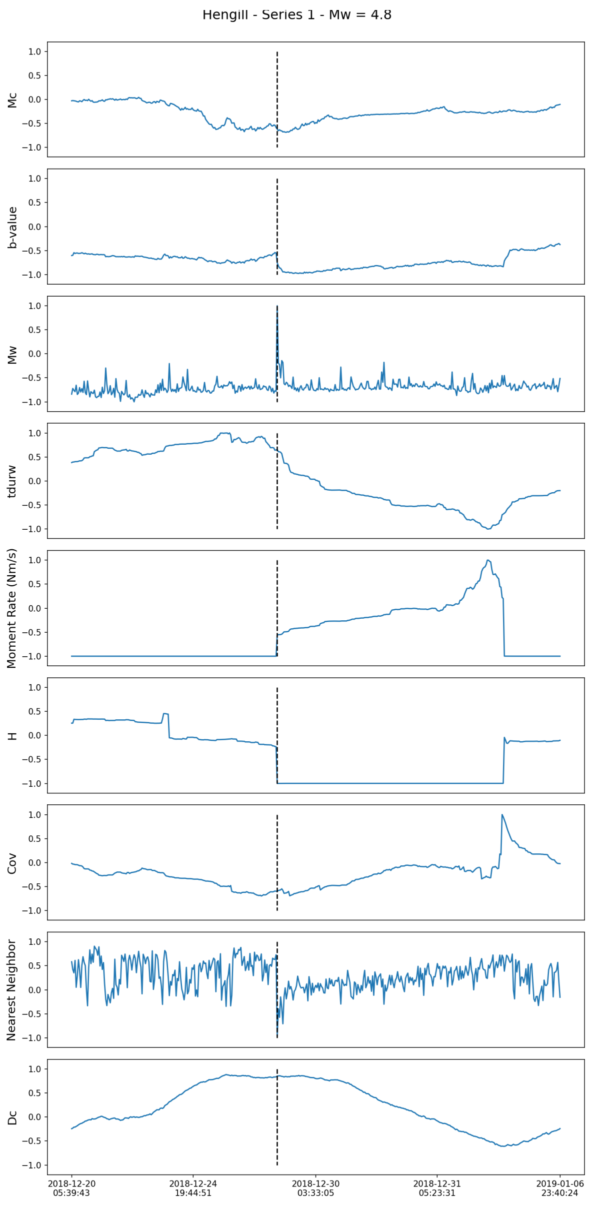
Hengill - Series 1 - Mw = 4.8
Mc
b-value
Mw
tdurw
Moment Rate (Nm/s)
H
Cov
Nearest Neighbor
Dc
2018-12-20 05:39:43
2018-12-24 19:44:51
2018-12-30 03:33:05
2018-12-31 05:23:31
2019-01-06 23:40:24

(n)

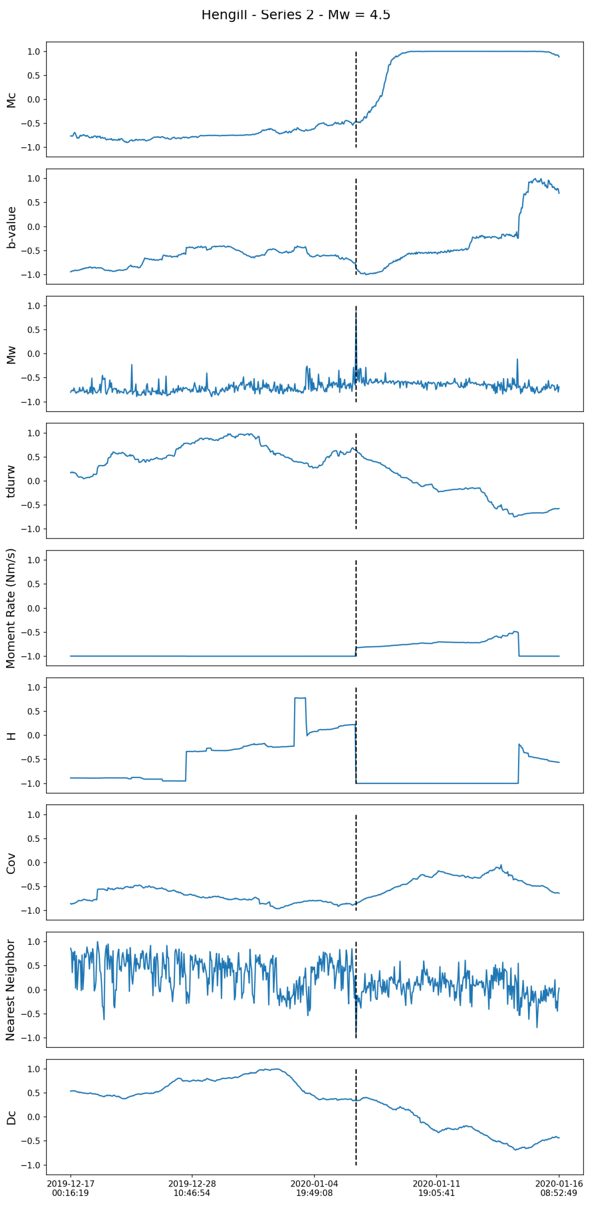
Hengill - Series 2 - Mw = 4.5
Mc
b-value
Mw
tdurw
Moment Rate (Nm/s)
H
Cov
Nearest Neighbor
Dc
2019-12-17 00:16:19
2019-12-28 10:46:54
2020-01-04 19:49:08
2020-01-11 19:05:41
2020-01-16 08:52:49

(o)

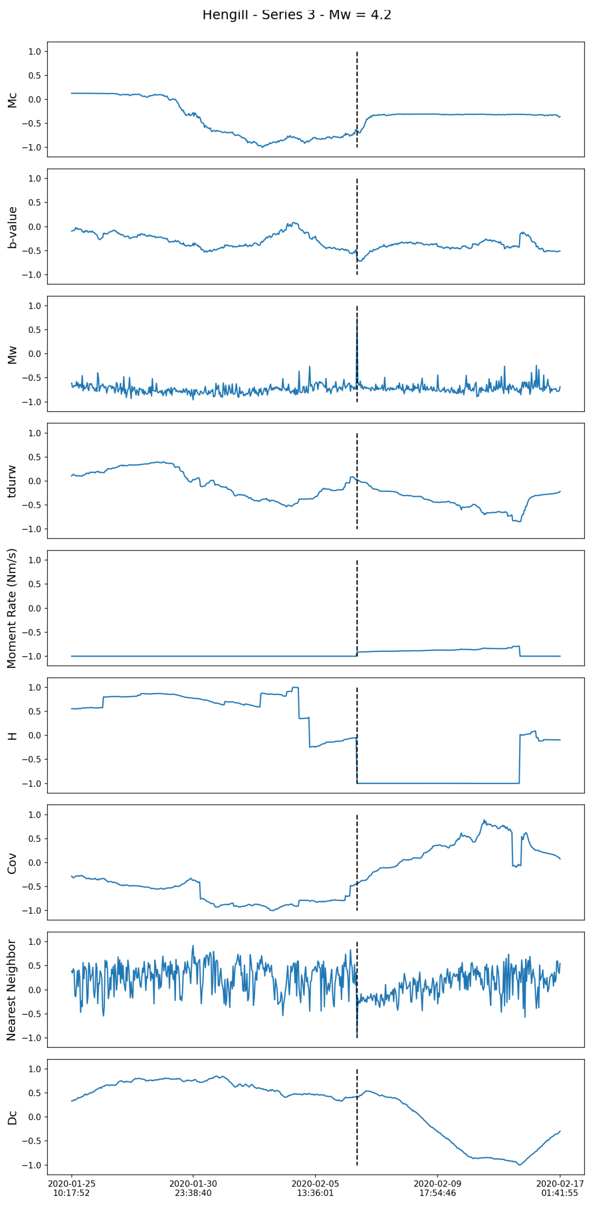
Hengill - Series 3 - Mw = 4.2
Mc
b-value
Mw
tdurw
Moment Rate (Nm/s)
H
Cov
Nearest Neighbor
Dc
2020-01-25 10:17:52
2020-01-30 23:38:40
2020-02-05 13:36:01
2020-02-09 17:54:46
2020-02-17 01:41:55

(p)

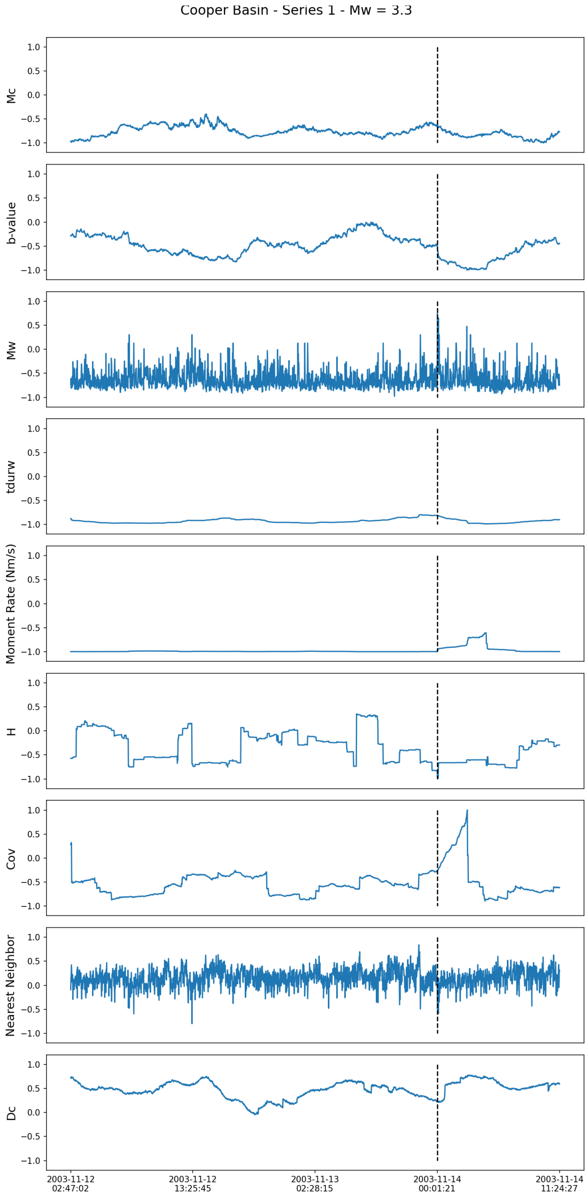

(q)

Figure S1. Features of the individual Series. Plot of the feature for each considered time series. On the x-axis there is the time. The dotted vertical line represents the largest event in the sequence whose magnitude is shown in the upper part of each panel. The features are normalized in the range [-1,1], separately for each region. From the top to bottom: minimum magnitude of completeness Mc, the b-value, moment magnitude (M_W), duration of events' group, moment rate, Shannon's Information Entropy, coefficient of variation CoV, Nearest-Neighbour distance, Fractal Dimension (Dc)

Further Details on Data splitting

The dataset under consideration, as reported in section "Study areas and data preparation" of the manuscript, is composed of 16 event series containing the event of interest for the present study in three different geothermal fields: The Geysers (TG), located in California (USA), Cooper Basin (CB), located in Australia, and The Hengill (HG), located in Iceland.
In particular, as for TG and CB geothermal fields, the collection of the samples considered for each of the highest magnitude events, i.e. what is defined as "time series" are composed of 2000 samples each, while for HG series of 600 samples have been extracted. The difference in the number of samples related to the last geothermal area is due to the fact that events collected in this catalogue are less frequent with respect to the other two catalogues. So, in order to make the time series span a reasonable temporal window, the length of the HG series, in terms of number of extracted samples, has been shortened.
In total 27800 samples $(2000 \times 8 + 2000 \times 5 + 600 \times 3)$ are present in the dataset. In particular, the samples composing each of the series are temporally consecutive: 1500 (TG and CB) or 350 (HG) events preceding the largest event in the sequence, the largest event itself and 499 (TG and CB) or 249 (HG) events after it constitute a single time series. It is worth underlining that this choice follows empirical and physical considerations, and the will to preserve a visual coherence between the phenomenon under investigation and the results reported in the manuscript. However it should be stressed that, since (as reported in the following and in the paper) the problem is framed as a classification one regarding each single sample, and so the presence of the largest event and subsequent events is unnecessary for the purpose of precursors recognition.
By using the dataset built as described before, a Train/Validation/Test split procedure has been conducted. In details, once taken apart three time series as Test set (i.e. 2000 for TG + 2000 for CB + 600 for HG samples related to three largest events), by considering the remaining 23200 samples, the 50% of them have been randomly selected to create a Training set while the remaining part has been selected as Validation set (as represented in the following figure).

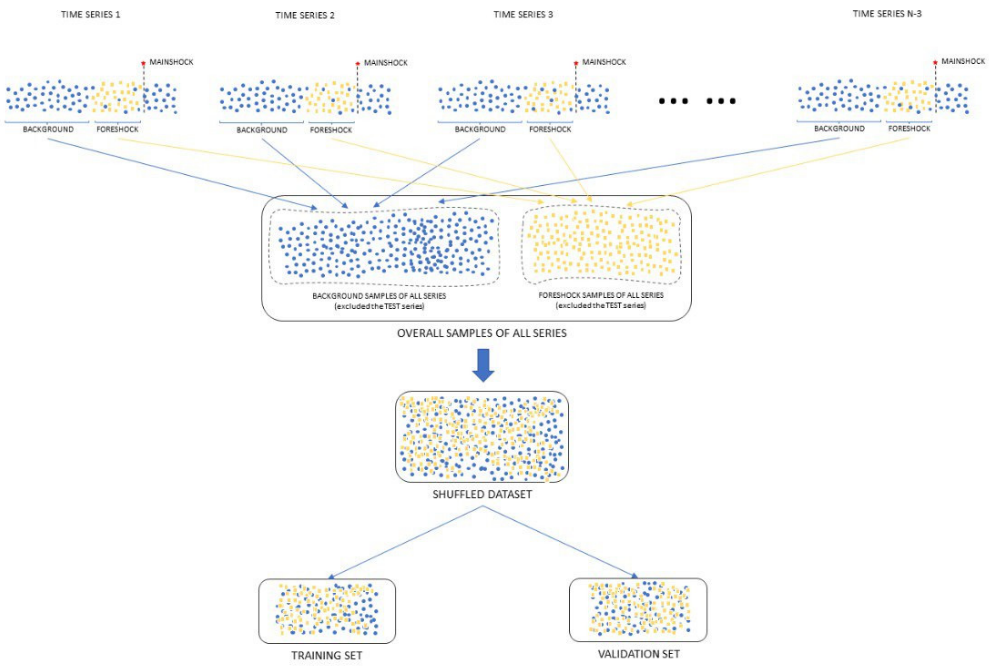

Figure S2. Data Splitting. Schematic representation of Train/Validation split.

Data Exploration and Discussion about Accuracy Results

From the splitting procedure described in the previous section it follows that, by construction, in the training set are present samples of both classes, background and precursors, for all the series considered, except the ones used as Test set. This implies that, as reported in the section "**PreD-Net** training" of the manuscript, even if the validation set is composed by samples unseen by the network during the training procedure, they belong to series, within the meaning of set of samples related to an event of interest , whose background events and precursors have been used to train the model: the regions in the features' space on which background and precursor validation samples lay, for each series, are identified during the training procedure. Therefore, we can assess that validation samples are obviously unknown by the network, but the contexts they are drawn from are somehow known.

On the other hand, since the samples used as Test set have been put apart before the Train/Validation split, both the single samples and the contexts are totally unknown in this case: the classification of these events is hence harder and relies on the generalization abilities of the model. These considerations impact the choice of preserving the natural disproportion of classes present in the collected dataset: drastically reducing the number of background points in order to balance the dataset can eventually cause the erroneous recognition of "background regions" in the features' space, degrading the model performance. The goal is then to train the model in such a way it recognizes as many patterns as possible, in order to also improve its generalization abilities.

The aforementioned discussion is supported by observations on a non-linear two-dimensional projection obtained through the TSNE algorithm of the features' vectors: for each samples' collection related to a event of interest (i.e. the largest magnitude event in a specific series), it has been found that projected samples distribute in small groups, which are generally coherent with respect to the labeling (see the following Figure S3). Moreover, it can be observed that groups of precursor samples are usually separated from ones belonging to the class background. This also justify the high prediction accuracy achieved on the Validation set: the **PreD-Net** Model is able to understand contexts from Training samples, i.e. it is able to understand, in the features' space, where small groups of background samples and of precursor samples are located. Validation samples, belonging to regions identified during the training phase, are then classified with an high degree of confidence due to the proximity to known regions.

The previous reasoning, of course, is not applicable to the Test samples, since the three series are completely unknown from both the point of view of the samples and the contexts: the PreD-Net model has to rely on its generalization capabilities, understanding, without any example, which "small clusters" are composed by background samples and which are composed by precursor ones. This translates into a much more complex task, that explains the drop of accuracy in the classification of samples whose contexts have been never seen in the training phase.

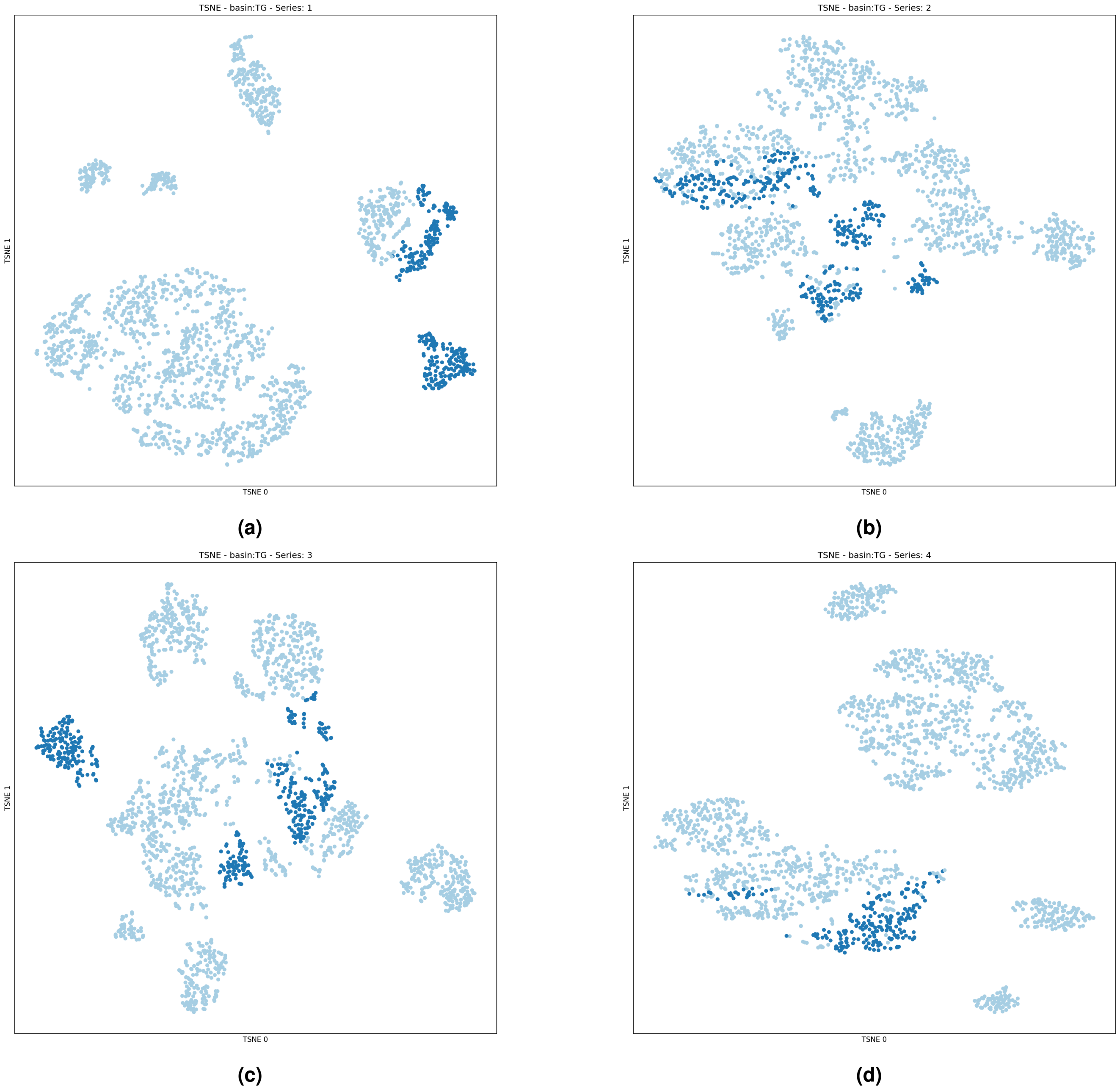
TSNE - basin:TG - Series: 1
TSNE 1
TSNE 0
TSNE - basin:TG - Series: 2
TSNE 1
TSNE 0
TSNE - basin:TG - Series: 3
TSNE 1
TSNE 0
TSNE - basin:TG - Series: 4
TSNE 1
TSNE 0

(a) **(b)** **(c)** **(d)**

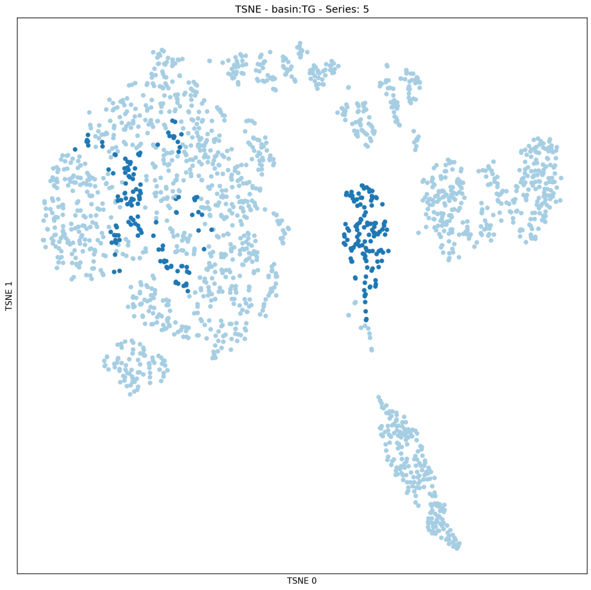
TSNE - basin:TG - Series: 5
TSNE 1
TSNE 0

(e)

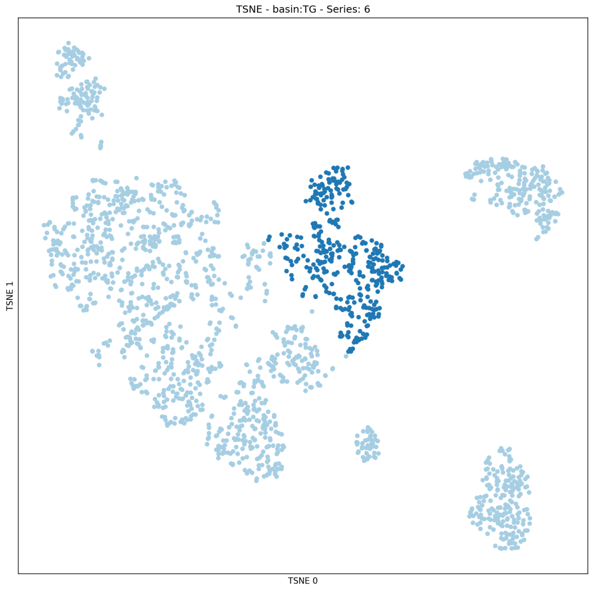
TSNE - basin:TG - Series: 6
TSNE 1
TSNE 0

(f)

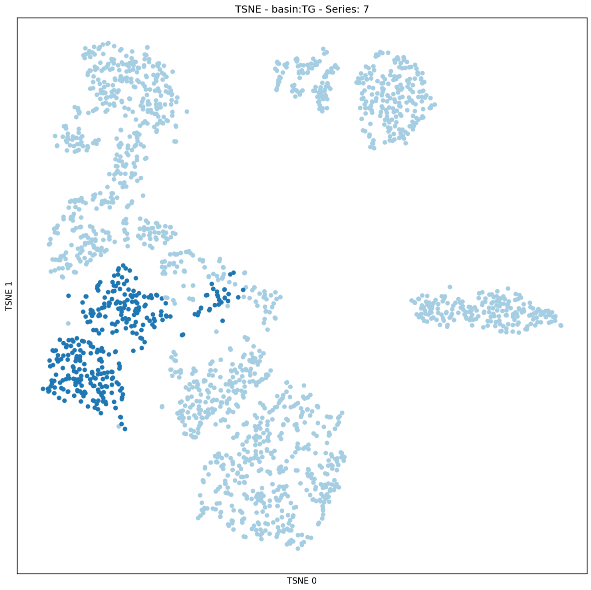
TSNE - basin:TG - Series: 7
TSNE 1
TSNE 0

(g)

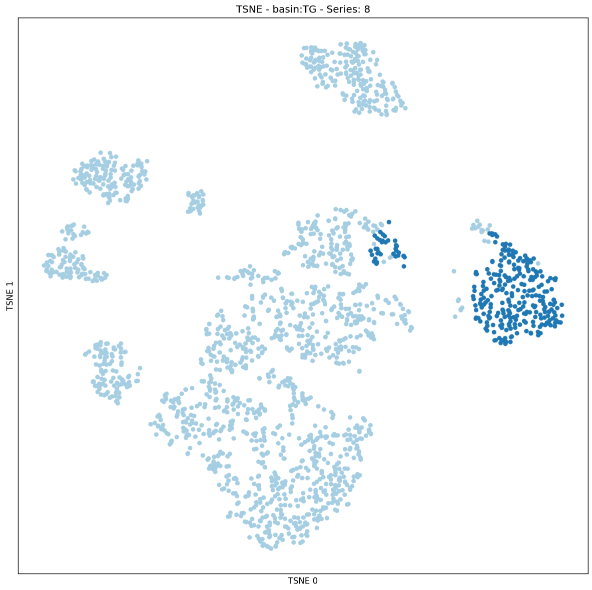
TSNE - basin:TG - Series: 8
TSNE 1
TSNE 0

(h)

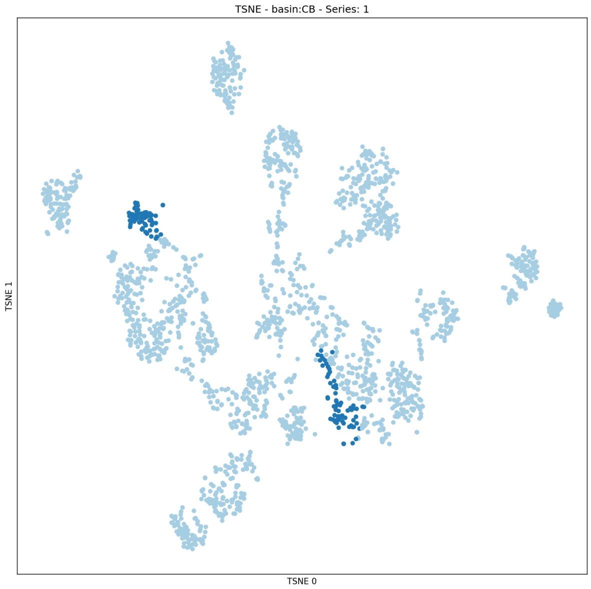
TSNE - basin:CB - Series: 1
TSNE 1
TSNE 0

(i)

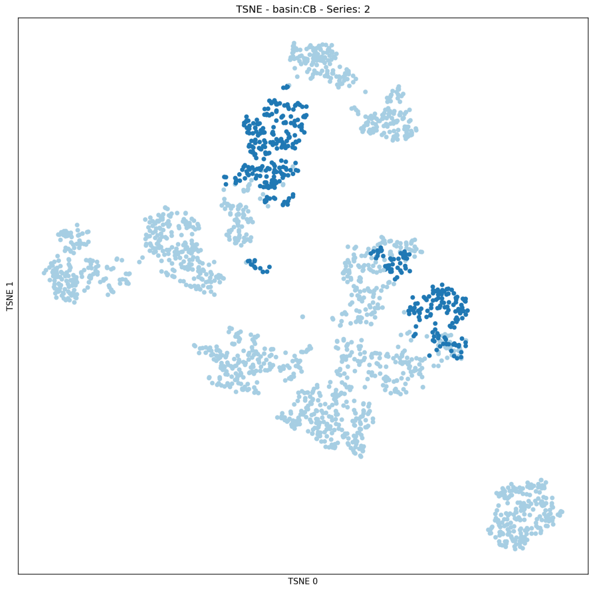
TSNE - basin:CB - Series: 2
TSNE 1
TSNE 0

(j)

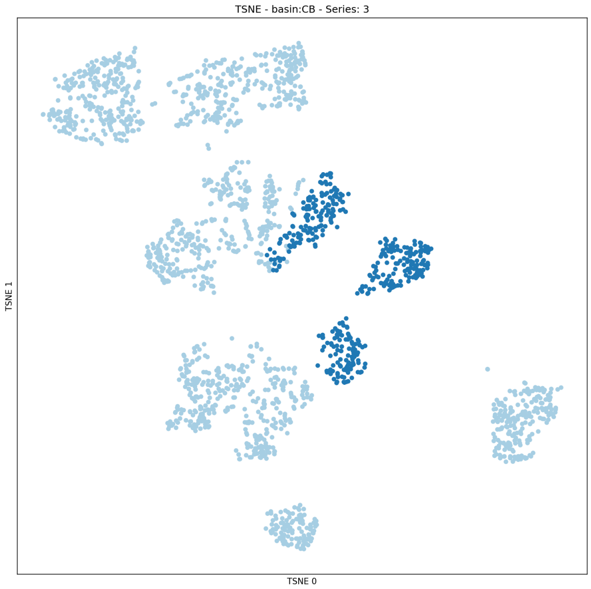
TSNE - basin:CB - Series: 3
TSNE 1
TSNE 0

(k)

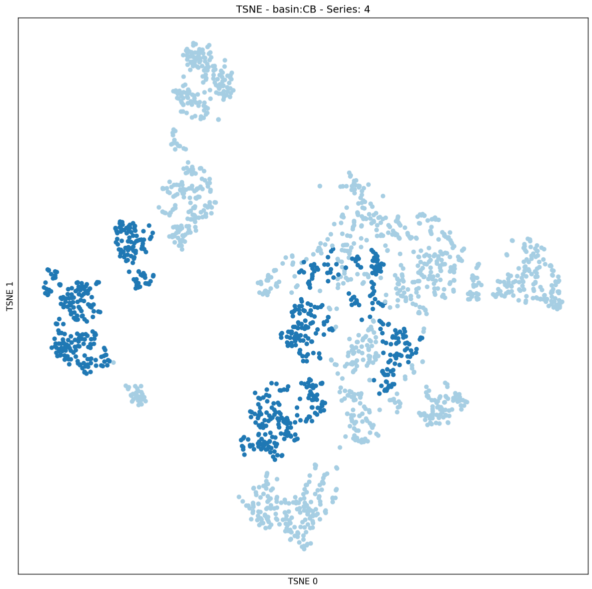
TSNE - basin:CB - Series: 4
TSNE 1
TSNE 0

(l)

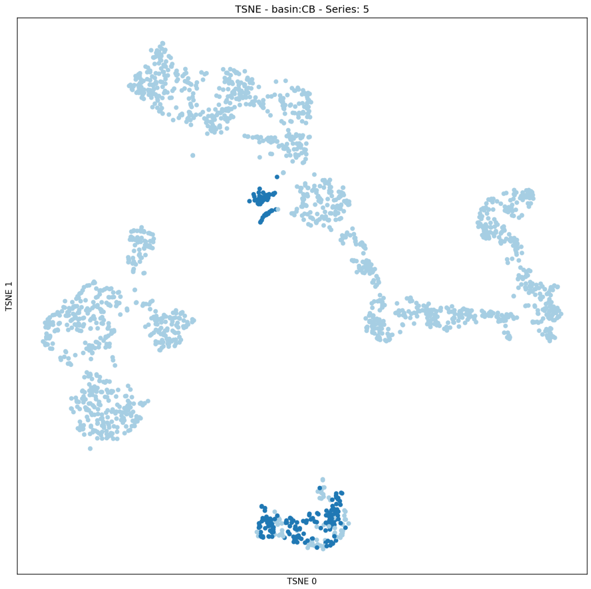
TSNE - basin:CB - Series: 5
TSNE 1
TSNE 0

(m)

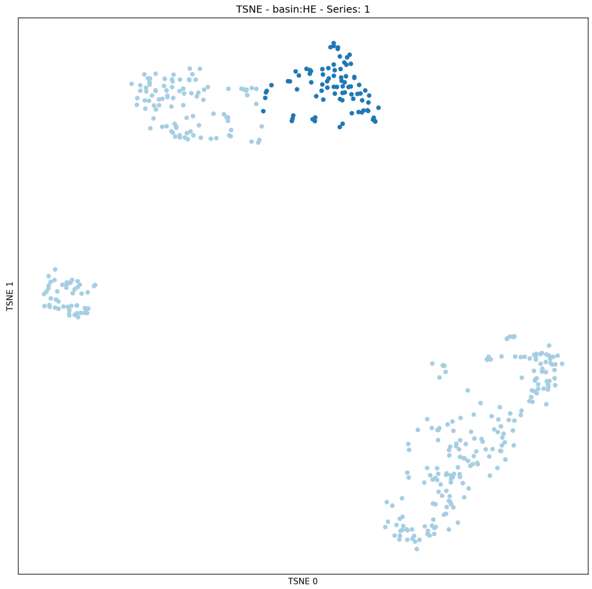
TSNE - basin:HE - Series: 1
TSNE 1
TSNE 0

(n)

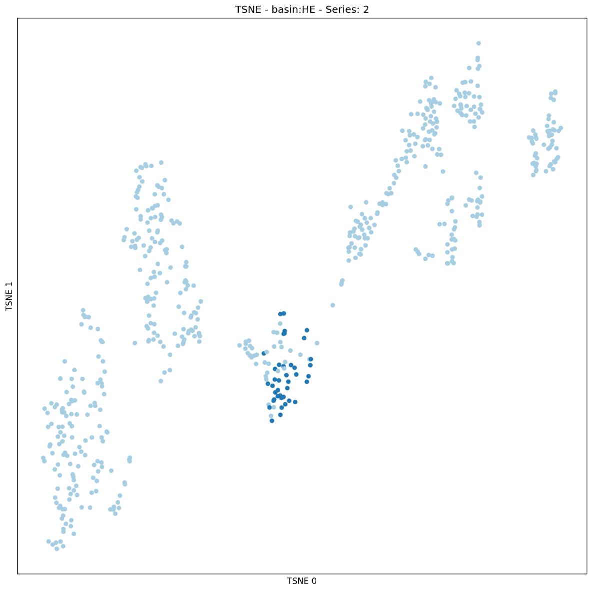
TSNE - basin:HE - Series: 2
TSNE 1
TSNE 0

(o)

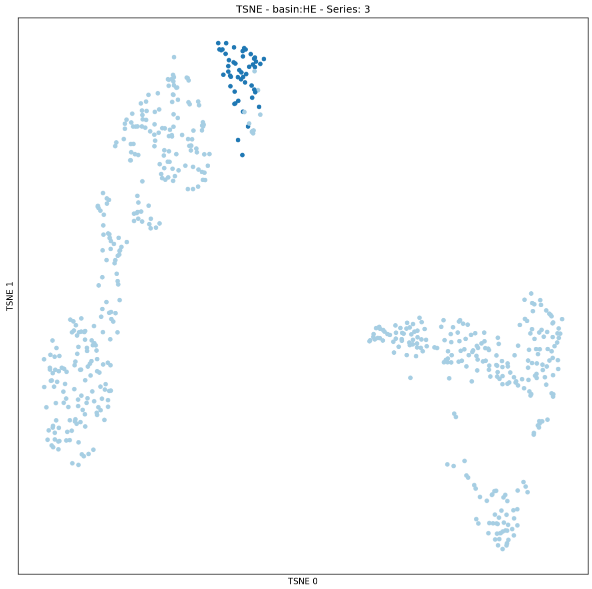
TSNE - basin:HE - Series: 3
TSNE 1
TSNE 0

(p)

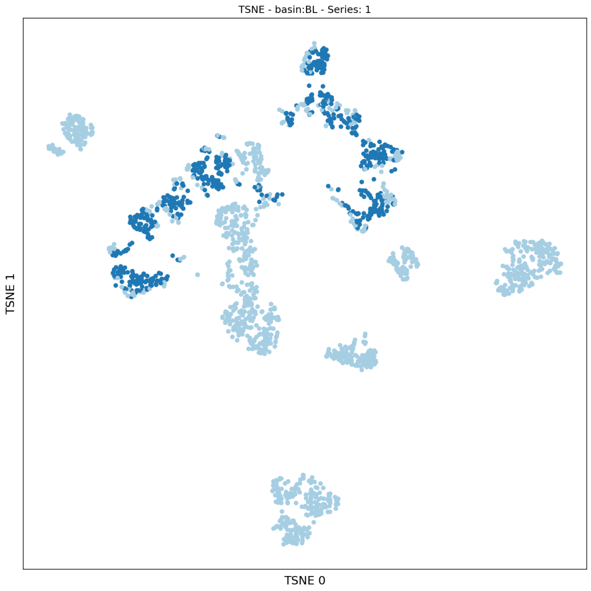

(q)

Figure S3. t-SNE. t-SNE representation of samples of the series. In light blue the background seismicity is reported, while precursory events are in dark blue.

Preliminary Investigation on Features' importance and Validation of PreD-Net Results

To assess the predictive importance of features, several tests have been carried out in order to verify that the PreD-Net model does not just recognize trivial patterns that could be extracted from the features taken individually. Since no obvious difference emerges among the features distribution in background and precursor contexts, as can be seen from the following Figure S4, statistical tests have been exploited to check whether background related features' distributions and precursors related ones differ in terms of mean, median or variance. In particular, once assessed the non-normality of the features, a Mann-Whitney test and a Levene test for the homoscedasticity have been explored.

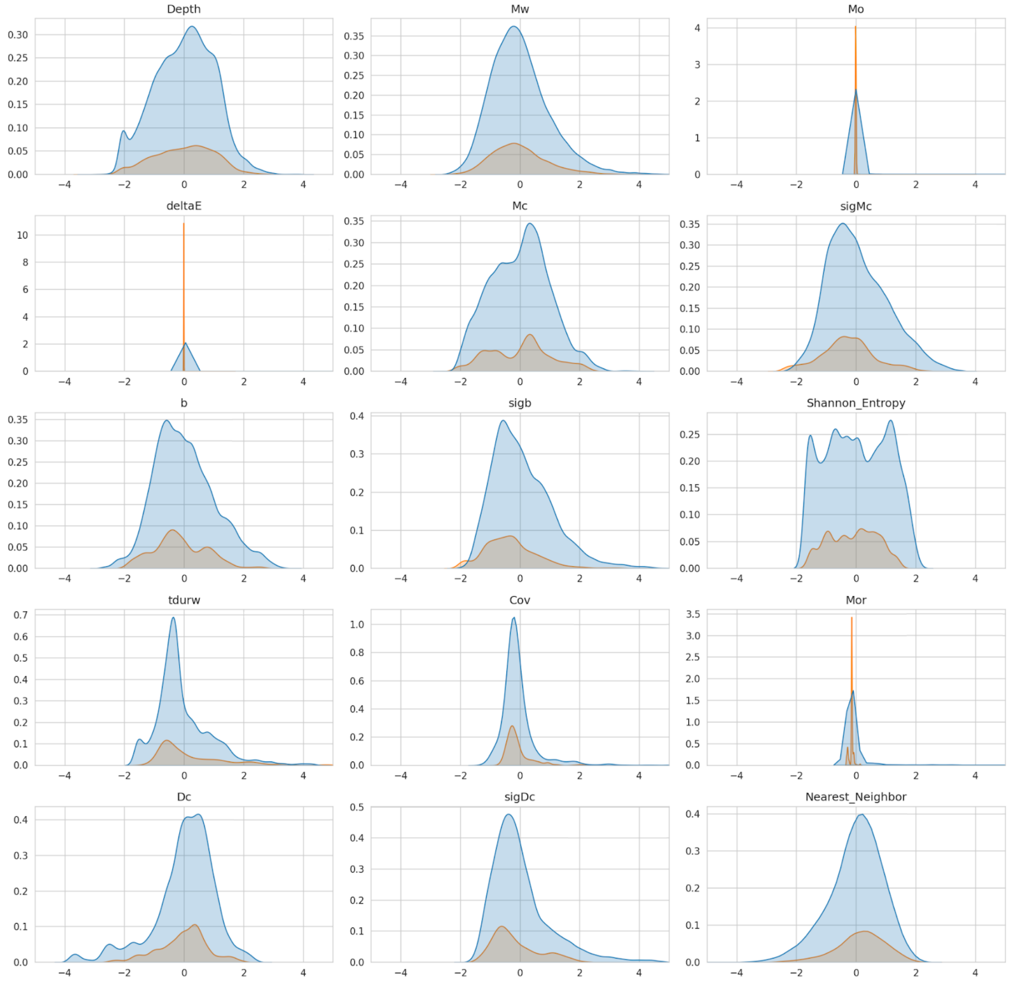

Figure S4. Data Distribution. Distribution plot of the features with respect to the two classes considered in the classification problem.

From the results shown in the Table S1, it emerges that some statistically relevant differences among features in the background and precursor contexts are present. However, no information about obvious patterns that connect a specific feature to the background/precursor label emerges.

Furthermore, in order to analyze the predictive power of the features, as well as the suitability of simpler algorithms with respect to the proposed PreD-Net for the task addressed in the manuscript, a 10-fold cross validation procedure has been implemented. In particular, the performances of some Machine Learning algorithms (Logistic Regression, Tree-based models, Support Vector Classification and a simple Multilayer Perceptron) have been compared with ones provided by PreD-Net, and a feature importance analysis has been conducted.

The cross validation has been then carried out on a dataset constituted by the entire data taken without the Test set. The predictive performances of the Machine Learning models have been evaluated, for each of the 10 training phases, both on the fold not used for the training, in this process named Validation set, and on the Test set, exploring the recognition abilities of the models about "known" contexts and its generalization strength.

In particular, the relevance of the single features has been investigated through a permutation importance strategy, i.e. randomly

Table S1. Mann-Whitney and Levene p value

Feature	Mann-Whitney - p value	Levene - p value
Depth	0.899	0.402
Mw	0.223	0.001
Seismic Moment	0.583	0.109
DeltaE	0.891	0.151
Mc	0.987	0.001
sigMc	0.002	0.001
b	0.002	0.002
sigb	0.001	0.003
Shannon Entropy	0.001	0.001
tdurw	0.003	0.003
Cov	0.074	0.002
Moment Rate	0.001	0.002
Dc	0.006	0.003
sigDc	0.001	0.001
Nearest Neighbour	0.004	0.001

shuffling the values of one feature at time during the iterations of the cross validation process: the accuracy variation of the models reflects the importance of the shuffled feature in terms of prediction. Repeating ten times such a procedure for each fold assures a robust estimation of the features' predictive power with respect to the applied model. The results concerning both the features' importance analysis and the peculiarity of PreD-Net performances are shown and discussed in Sec. "Investigation on Features' importance" and Sec."Investigation on Model Performances " of the manuscript.
As concern these last set of tests, to further assess the reliability of the obtained high performances , a corrected paired t-test[1] has been carried out to verify that the differences of mean accuracy metrics between the models are statistically significant.

Table S2. p-values from the t-test

	Logistic Regression	Random Forest	XGBoost	Bagged Extremely Randomized Tree	MLP
Accuracy	0.0	0.0	0.000013	0.0	0.0
F1 Score	0.0	0.0	0.0	0.0	0.000015
ROC AUC	0.0	0.000011	0.000002	0.0	0.0

In the Table S2, p-values from the t-test computed on evaluation metrics obtained in the 10 fold cross validation procedure on the Test set are shown. This null-hypothesis test confirms what has been discussed before, providing supplementary evidence on the peculiarity of the results obtained through the PreD-Net architecture.

Further Details of PreD-Net Architecture and Training Procedure

The PreD-Net model consists of a three branch network composed of a Convolutional (CNN) stack, a Dilated convolutional (DiCNN) stack and a recurrent (GRU) stack. The architecture has been designed to operate with or without lagged variables associated to each sample: the first refers to the case each sample is taken individually (each sample will be a vector 1×16), the latter refers to the case in which temporal subsequences are taken into account, i.e. the case that features' values of events $(t-m)-(t-1)$ are associated to the generic sample at timestep t (the sample will be a vector $1 \times (m \times 16)$, with m representing the length of the subsequence considered, i.e. m timesteps).
In detail, the central branch of the network follows a structure of shrinking convolutional one dimensional filters and expanding transposed one dimensional convolutional filters. This architecture performs pattern extraction and noise reduction along the features' dimension, therefore acting as an Autoencoder.
The latent space generated by the encoding stage of the central branch serves as input of a stack of GRU layers, whose aim is to recognize temporal patterns between the sample under consideration and related lagged ones, if present. In other words, given an input vector $1 \times (m \times 16)$ which represents a sample at time t accompanied by its $(m-1)$ preceding events, the latent space generated by the Encoding section of the central branch will be a vector $m \times nF$, with nF being the number of convolutional filters; this will be the input of the GRU stack.

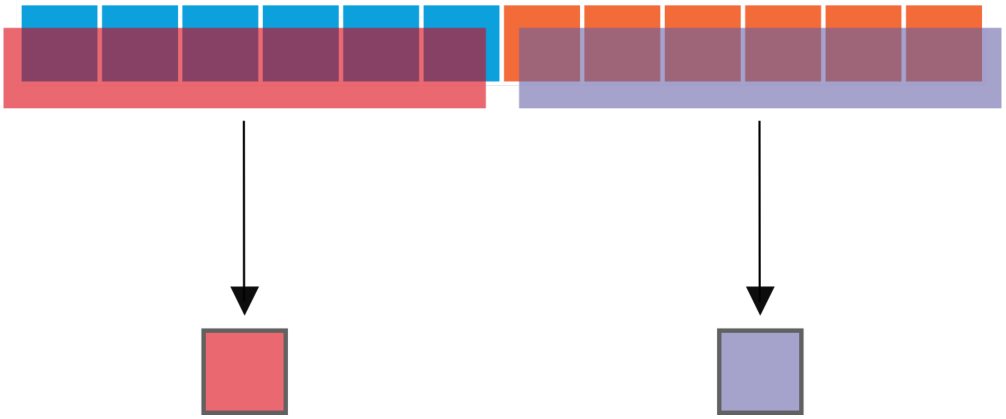

Figure S5. Convolutional Filters. Example of how convolutional filters work in the central branch of the network: given 2 events, with the related features (blue squares for the first sample, orange ones for the second) convolutional filters act in the way the variables related to a single sample are compressed to a low-dimensional representation.

The third branch, composed by dilated convolutional layers, acts on the input of the network extracting temporal patterns among the single features: due to a dilation rate of 16 (the number of features), these convolutional filters act on the temporal stack of a single feature at a time. The aim of this branch is therefore to "understand" the behavior, in time, of each of the single variables out of the 16, returning an output of dimensions $16 \times nF$, where also in this case nF represents the number of filters associated to this convolutional stack.

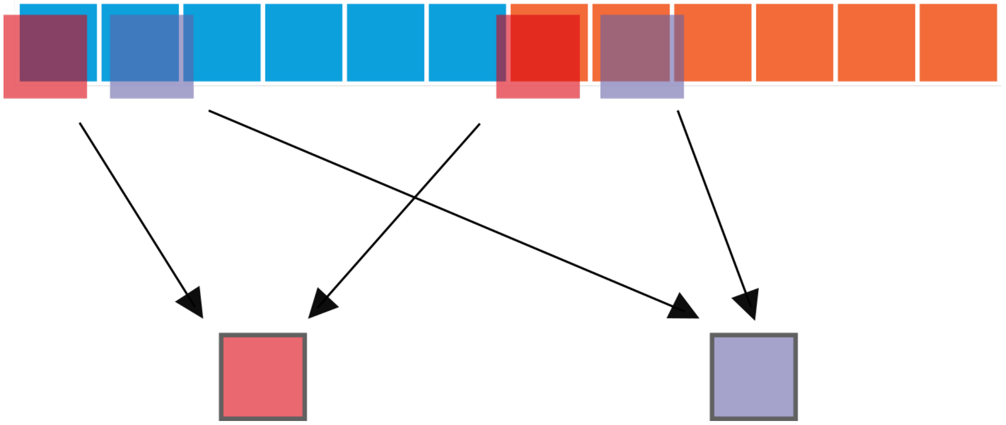

Figure S6. Dilated Convolutional Filters. Example of how dilated convolutional filters work in the network: given 2 events, with the related features (blue squares for the first sample, orange ones for the second) dilated convolutional filters act in order to extract patterns between different timestep of a specific feature at time. In the case no lagged variables are provided, this branch acts as an embedding.

The main idea behind the designed architecture has been to analyze the information in input from different points of view, providing a compressed representation of the input data and analyzing temporal relations between events and single features. As aforementioned, the model has been prepared to work with temporal subsequences of events as well as a single event at time. All the performed experiments have reported better classification performances in the second case: this could be explained with the fact that some of the features are computed on backward temporal windows that implicitly express temporal patterns; on the contrary, taking into account samples added with lagged values introduces a degree of complexity due to the choice of lagged timesteps to consider, therefore introducing noise in the training process. The results shown in the manuscript refers to the classification of single events. In this case, obviously, the GRU branch of the network is cut off, while the dilated convolutions act as an embedding for each single feature of the sample.
The choice of preserving the architecture of the neural network with also the GRU branch has been made to keep the possibility of applying the model to new datasets exploiting also temporal subsequences: as regards the three catalogs tested in the presented work, and as discussed before, the performances about the classification task suggest the "single event at time" shape

for the input data. However, it should not be excluded that on different catalogs, or considering more data, i.e. more time series contexts, the contribution of temporal patterns among events overcome the introduced noise, improving the overall accuracy of the classification.
As pointed out in the section "Data Exploration and Discussion about Accuracy Results" of these supplementary materials, the dataset presented in the manuscript suffers from a certain degree of class imbalance: while the choice of preserving this characteristic has the aim of keeping a physical peculiarity of the problem, this turns the classification task into a rare case recognition task, which may require specific strategies to be addressed.
The loss function adopted in the case under examination is a combination of Binary Categorical Cross-Entropy and Dice Coefficient on the class 1, i.e. precursors. This combination aims to exploit the stability of the former with respect to the backpropagation process while compensating the class unbalance through the latter, which acts as a penalization term on misclassified samples belonging to the minority class. Defined the Binary Crossentropy as:

$$CE(p,q) = -p \cdot \log(q) + (1-p) \cdot \log(1-q)$$

and the Dice Coefficient, written as loss, as:

$$DICE(p,q) = 1 - \frac{2\sum pq}{\sum p^2 + \sum q^2}$$

the loss function exploited in the experimental phase presented in the work can be written as:

$$Loss = (1-\beta) \cdot \bar{CE} + \beta \cdot DICE_1$$

where β acts as a balancing parameter. The determination of the value for such a balancing parameter has been carried out in the training process itself: β has been defined as a trainable parameter, and its value has been optimized during the optimization of the loss function. However, since the Dice component constitutes a corrective term for the Binary Cross Entropy and must not overcome this component, the value of β has been bounded in the interval $[0.2, 0.5]$.
Moreover, as pointed out in the introductory section of the manuscript, the labeling of precursors require empirical/subjective criteria to be carried out; to take into account the uncertainty connected to such criteria, as well as to allow the model to better generalize over the discrimination of backgrounds and precursors, a relaxation parameter for the crossentropy has been introduced.
Finally, in order to enhance the network performances, a specific training procedure has been developed: subsequent training sub-phases of the network are carried out with finer learning rates, in such a way each sub-phase starts from the best result (in terms of value of the loss function, and related setting of the network's weights) attained in the preceding one. In particular, each sub-phase ends when an early-stopping criteria is matched; then, best weights of the network are restored, and a new sub-phase starts with a new learning rate obtained as a fraction of the previous one. The training procedure ends when a limit value for the learning rate is reached. This procedure ensures that the minimization process over the loss function does not get stuck in local minima, and stabilizes the performances of the PreD-Net with respect to the uncertainty introduced by the random shuffling of the Train/Val split and the random initial allocation of the layers' weights. In the Table S3 main parameters for the network, training phase and loss function are reported.

Further Test Experiments

The process described in section "PreD-Net training and prediction" of the manuscript has been carried out considering other time series as Test set. The following figures show the obtained results.
Moreover, an experiment has been carried out to test the sensitivity of the settings used for the warning strategy. In the following time series of 2000 elements have been extracted from the TG catalogue, in particular chosen to not contain any events of magnitude greater than 2.1. In other words, PreD-Net has been tested on a series of pure backgrounds in order to assess its performances. As can be observed, for each series, the whole sequence is correctly predicted as background. Furthermore, it can be noticed that the developed warning strategy correctly returns no alert since it is based on the slope of cumulative predicted probability for the precursors: in the regions this probability suddenly increase, a peak of the CDF derivative is present; however, since no threshold is crossed, it remains green due to the smoothness of the CDF and the low probability of being precursors for the large majority of considered samples.

Table S3. Parameter Values for PreD-Net Training

Parameters	Value
**Architectural Parameters**	
# Convolutional Filters	32
GRU Layers	[64, 64, 64]
Dense Layers	[512, 64, 8]
**Learning Parameters**	
Optimizer	Adam
Initial Learning Rate	$1e-3$
Decreasing Factor for LR	3
Final Learning Rate	$1e-6$
L2 Regularization	$2e-6$
# Epochs	1000
Initial Patience	50
Increasing Factor for Patience	0.2
**Loss Parameters**	
Relaxation Parameter	0.025
Bounds for the Balancing Coefficient	(0.2 - 0.5)

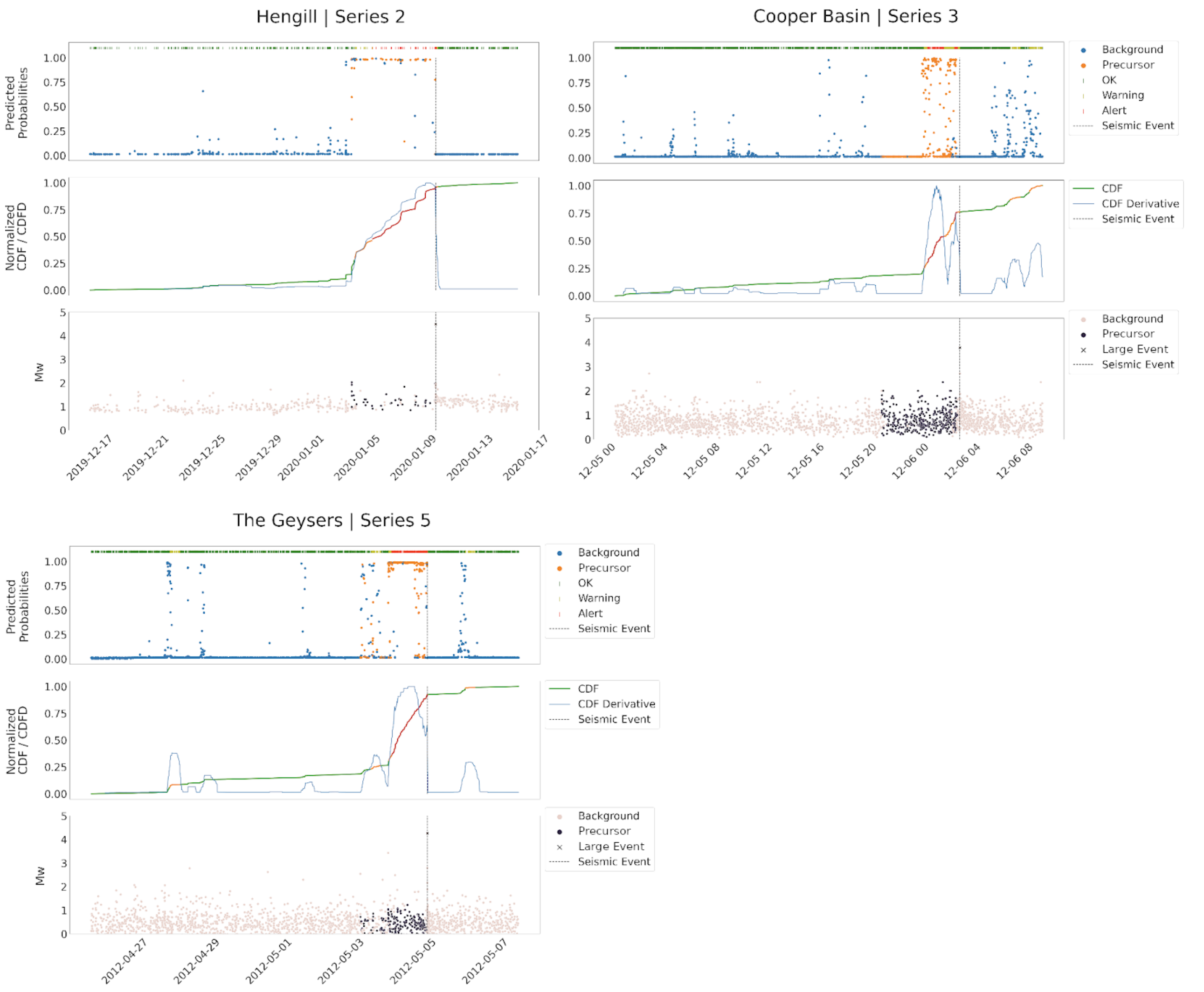

Figure S7. Test results of PreD-Net: Test of PreD-Net by considering, as Test set, the series 2 of the Hengill geothermal region, the series 3 of Cooper Basin and the series 5 of The Geysers.In the upper panel of each subfigure is reported the probability an event is predicted as "precursor" (y-axis), while the color represents the ground truth (orange for precursors, blue for background samples). In the middle panel is reported the warning strategy. In the last panel values of M_W for the samples considered are reported, in pink if background samples, in dark if precursors.

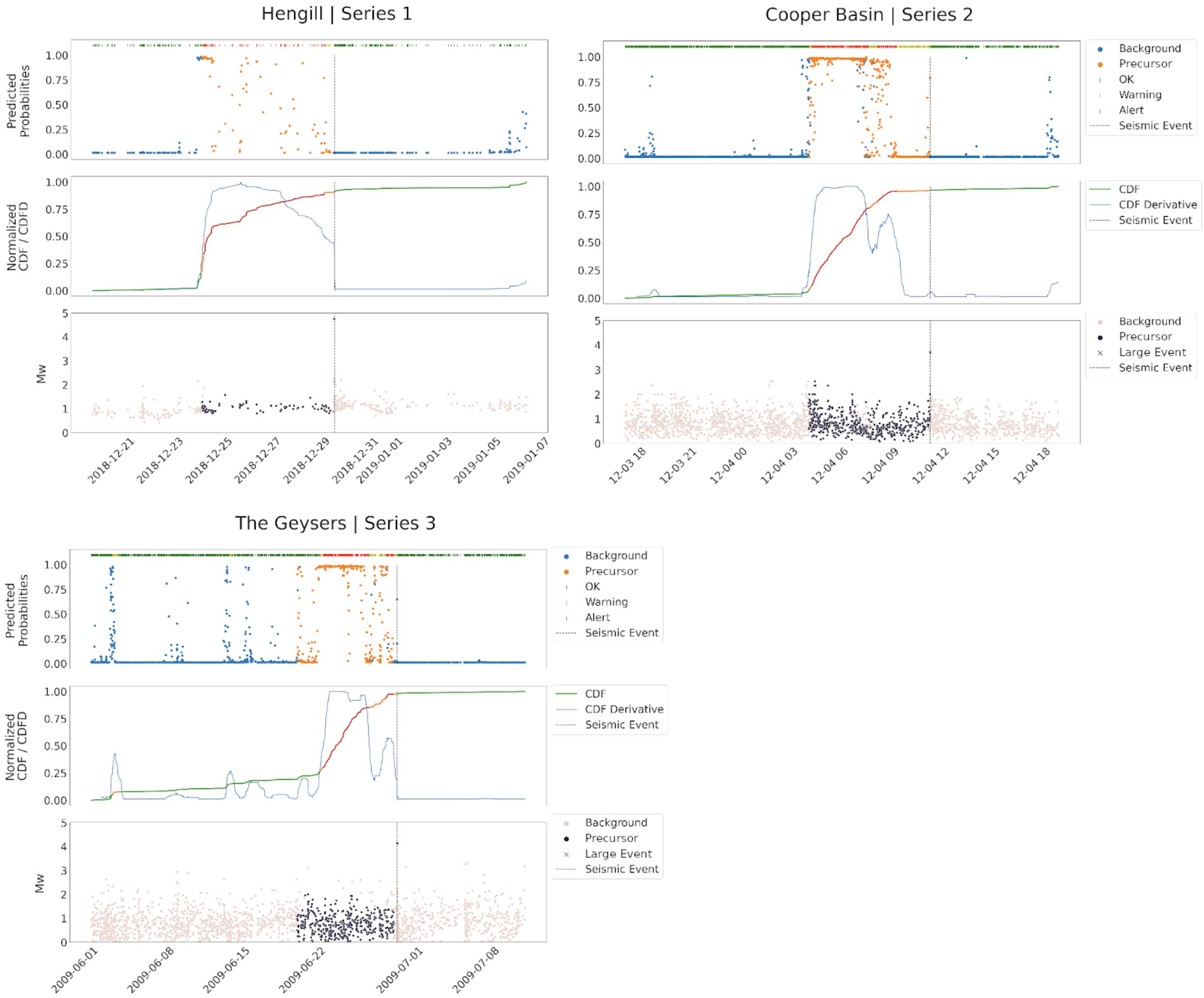

Figure S8. **Test results of PreD-Net**: Test results considering, as Test set, the series 1 of the Hengill geothermal region, the series 2 of Cooper Basin and the series 3 of The Geysers.In the upper panel of each subfigure is reported the probability an event is predicted as "precursor" (y-axis), while the color represents the ground truth (orange for precursors, blue for background samples). In the middle panel is reported the warning strategy. In the last panel values of M_W for the samples considered are reported, in pink if background samples, in dark if precursors.

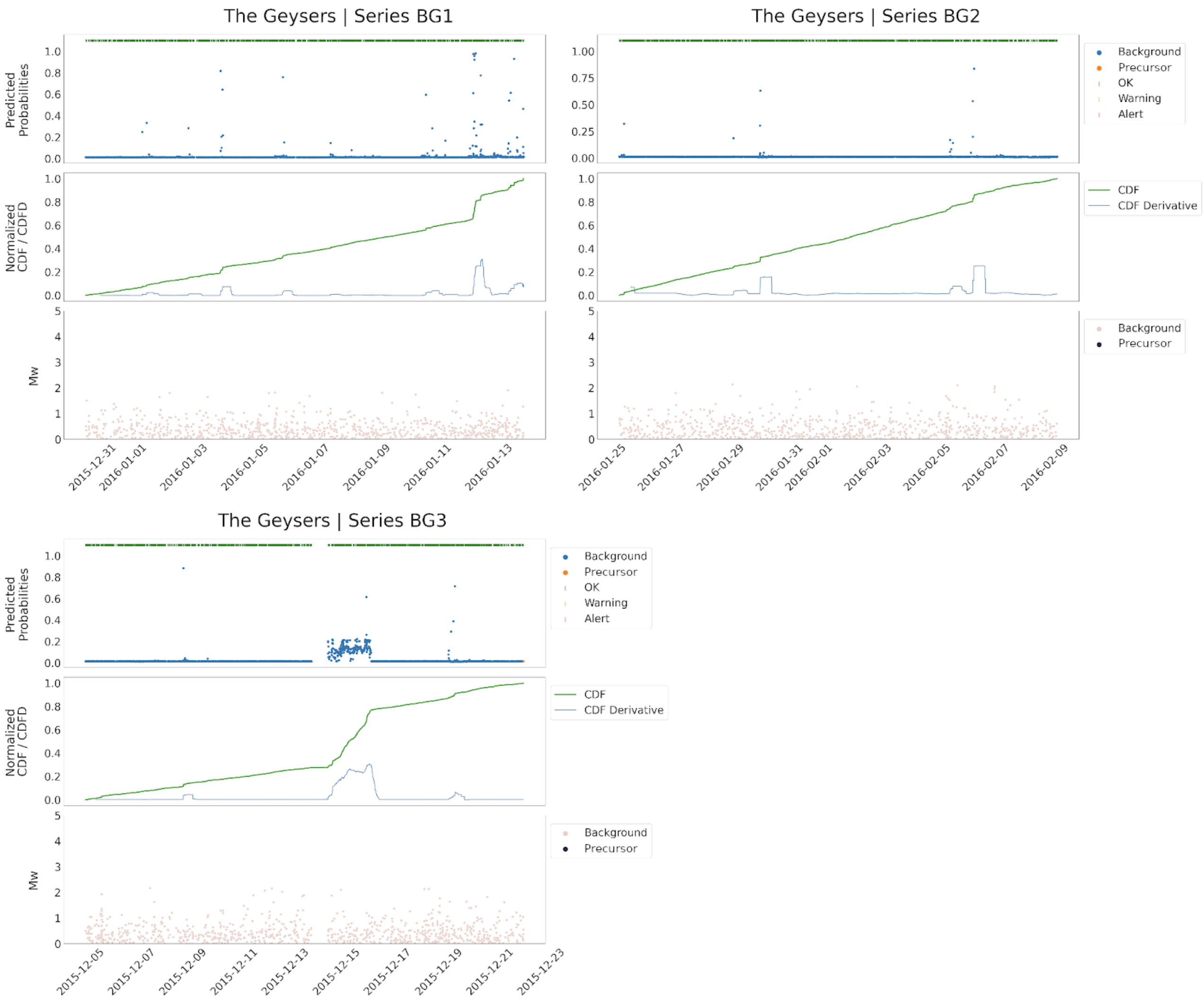

Figure S9. Test results on Background: Test results of PreD-Net considering series of only background samples taken from TG. It is worth noticing that, for visual purposes, the CDF and the CDCF are normalized on the 2000 samples constituting the whole time series.

References

1. Nadeau, C. & Bengio, Y. Inference for the generalization error. *Mach. Learn.* **52**, 239–281 (2003).
